# Four selenoprotein P genes exist in salmonids: Analysis of their origin and expression following Se supplementation and bacterial infection

**DOI:** 10.1371/journal.pone.0209381

**Published:** 2018-12-20

**Authors:** Moritz A. N. Pohl, Tiehui Wang, Thitiya Pohl, John Sweetman, Samuel A. M. Martin, Christopher J. Secombes

**Affiliations:** 1 Scottish Fish Immunology Research Centre, Institute of Biological and Environmental Sciences, University of Aberdeen, Aberdeen, United Kingdom; 2 Alltech, Springcroft, Mosshill, Brora, United Kingdom; University of Idaho, UNITED STATES

## Abstract

The following research was conducted to elucidate the evolution and expression of salmonid selenoprotein P (SelP), a selenoprotein that is unique in having multiple selenocysteine (Sec) residues, following supranutritional selenium supplementation and infection in rainbow trout. We show that in salmonids SelP is present as four paralogues and that the diversification of SelP genes during vertebrate evolution relates to whole genome duplication events. With 17 and 16 selenocysteine residues for rainbow trout (*Oncorhynchus mykiss*)/Atlantic salmon (*Salmo salar*) SelPa1 and SelPa2 proteins respectively and 1 or 2 (trout or salmon) and 4 or 3 (trout or salmon) selenocysteine residues for salmonid SelPb1 and SelPb2 proteins respectively, this is the highest number of (predicted) multiple selenocysteine containing SelP proteins reported for any vertebrate species to date. To investigate the effects of selenium form on SelP expression we added different concentrations (1 nM– 10 μM) of organic or inorganic selenium to a trout cell line (RTG-2 cells) and analysed changes in mRNA abundance. We next studied the impact of supplementation on the potential modulation of these transcripts by PAMPs and proinflammatory cytokines in RTG-2 and RTS-11 cells. These experiments revealed that selenium type influenced the responses, and that SelP gene subfunctionalisation was apparent. To get an insight into the expression patterns *in vivo* we conducted a feeding trial with 2 diets differing in selenium content and 5 weeks later challenged the trout with a bacterial pathogen *(Aeromonas salmonicida*). Four tissues were analysed for SelP paralogue expression. The results show a significant induction of SelPa1 in gills and intestine following infection in selenium supplemented fish and for SelPa2 in gills. SelPb1 was significantly reduced in head kidney of both diet groups following infection, whilst SelPb2 was significantly upregulated in skin of both diet groups post infection. Overall these findings reveal differential expression profiles for the SelPa/SelPb paralogues in trout, influenced by selenium supply, cell type/tissue and stimulant. The increase of multiple Sec containing SelP proteins in salmonids could indicate an enhanced requirement for selenium in this lineage.

## Introduction

Selenium (Se) is present in two forms in eukaryotic proteins- the rare amino acid selenocysteine (Sec) and selenomethionine (SeMet). The term selenoprotein is used exclusively for proteins containing Sec residues since this is the main biologically active form of selenium in proteins. Sec is encoded by the triplet codon UGA that is usually interpreted as a stop codon during translation. The inclusion of Sec into the polypeptide chain of proteins is achieved by a specific molecular insertion machinery including a Sec insertion sequence (SECIS), SECIS-recognizing proteins, a specific elongation factor and a Sec-loaded tRNA. The SECIS element is present as a stem-loop in the 3´-untranslated region (UTR) of the selenoprotein mRNA [1].

The human genome has 25 annotated genes to encode for selenoproteins [2]. Selenoprotein P (SelP) is one of these and was first discovered in the early 1970´s when a protein was detected incorporating injected ^75^Se in rat plasma [3], that was distinct from the known plasma selenoprotein glutathione peroxidase (GPx) and accounted for 60% of plasma Se levels [4,5]. It is unique within this class of proteins in having multiple Sec residues, with human and mouse SelP both having 10 [6]. Mouse SelP ^-/-^ knock-out mutants are embryonically non-lethal in contrast to other selenoprotein deletions in the mouse model, as seen with GPx4, thioredoxin reductase 1 (TRxR1), TRxR3 and the selenocysteine tRNA [7,8]. However, the animals show reduced motor coordination from 3 weeks after birth and reduced growth/ weight gain after 5 weeks, with reported sporadic fatalities [9]. Studies of a further (independent) mouse SelP ^-/-^ knock-out line showed that Se levels were significantly decreased in plasma, brain and kidney, and only liver Se levels were significantly elevated [10]. Male fertility was also sharply reduced. These findings of altered Se distribution within an organism are evidence for a role of SelP as a Se storage and transport protein, albeit probably not the only Se transport mechanism *in vivo*. However, SelP also functions as an antioxidant, making it a selenoprotein with a dual function [11]. The uptake of hepatically derived SelP by other cells takes place by receptor-mediated endocytosis [12–14] and an N-terminal heparin binding site has been demonstrated [15].

In the zebrafish, *Danio rerio*, two paralogues of the mammalian protein have been discovered, with one containing 17 Sec and one having a single Sec, and were termed SelPa and SelPb respectively [6]. Both are similarly divergent but there was a human SelP-zebrafish SelPa linkage identified [6], suggesting these could be orthologues. Homologues of the zebrafish SelPs have also been cloned from rainbow trout, *O*. *mykiss*, in our lab and a 406 amino acid (aa) protein containing 17 Sec and a 279 aa protein containing a single Sec were predicted from the obtained mRNAs and their expression confirmed [16]. Initially it was not clear whether these genes could have been generated by the teleost specific whole genome duplication (WGD) event (TGD), that occurred at the base of this vertebrate group (Pasquier et al. 2017). However, SelPb has now been discovered in a broad range of vertebrate species and arose early during vertebrate evolution and has apparently been lost in placental mammals (with the exception of armadillo) [17,18]. In addition to the TGD, the salmonid lineage has undergone a further WGD [19], and is in the state of rediploidization [20,21]. With this genomic background salmonids potentially possess sets of paralogs not present in other teleost lineages. Given the importance of selenium for the organism and its role as an immunostimulant albeit potent toxin at higher concentrations, an additional set of Selenoprotein P genes would make a remarkable trait that is unique among vertebrates. Due to the Sec codon peculiarity, new selenoproteins easily evade detection in computational genome annotation pipelines despite being intriguing proteins with versatile functions although often incompletely understood or unknown.

In the present study we first undertook a search for additional SelP paralogues in salmonids, and confirmed that indeed additional genes exist, that we termed SelPa2 and SelPb2. We then examined whether the salmonid paralogues were derived from the TGD or the salmonid specific WGD (or some other means), by examination of the SelP loci throughout vertebrates. Whether the paralogues had diverged functionally was next studied, by undertaking a transcriptional analysis in rainbow trout cell lines after *in vitro* addition of two forms of Se and following stimulation of the cells with cytokines or PAMPS. Lastly, since the role of selenium during immune responses is mainly limited to mammalian studies and little is known about the health benefits of dietary Se in aquaculture, we studied the potential modulation of the paralogues in trout given a diet supplemented with Se. Functional feeds such as these provide a promising approach to influence fish disease resistance, welfare and fillet quality. To produce the diet Sel-Plex, a mainly organic form of Se yeast, was added to achieve levels above those currently present in commercial diets. Following feeding for 5 weeks a subset of the trout were infected with a bacterial pathogen and the impact on SelP paralogue expression in the Se supplemented fish assessed.

## Materials and methods

### Bioinformatics analysis

The known salmonid SelPa and SelPb was used to Blast search [22] against the expressed sequence tag (EST) database of NCBI. Returned hits were aligned and consensus sequences built. These consensus sequences were initially aligned against genomic scaffolds of rainbow trout (*O*. *mykiss*) and Atlantic salmon (*S*. *salar*), leading to the prediction of a second SelPa and SelPb. Further analysis were performed on the recent released trout (GCA_002163495.1, Omyk_1.0) and salmon (GCA_000233375.4, ICSASG_v2) genome assembly. We renamed the known salmonid SelP genes as SelPa1 and SelPb1 and the new ones as SelPa2 and SelPb2, respectively. The presence and structure of SECIS elements was predicted with the selenoprotein prediction server [23] at http://seblastian.crg.es/. Phylogenetic tree analysis on the protein sequences was performed with MEGA7 [24] using the neighbour-joining strategy and the Jones-Taylor-Thornton (JTT) model, with 10,000 generations of bootstrapping were undertaken. Alignments were performed with the MAFFT online service [25]. The information of syntenic blocks for each gene/locus was extracted from NCBI reference genomes (*O*. *mykiss*: GCF_002163495.1 & *S*.*salar*: GCF_000233375.1) at ensembl.org and the annotated salmon genome as published at SalmoBase (salmobase.org). Overall SelP sequence identities and similarities were calculated using MatGAT2.01 [26]. The visualisation of the intron-exon structure was performed using the IBS software tool [27].

### Sequence confirmation of predicted salmonid SelPa2 and SelPb2 genes

The prediction of salmonid SelPa2 and SelPb2 sequences was confirmed in both rainbow trout and Atlantic salmon by polymerase chain reaction (PCR) cloning using primers designed at the 5’-untranslated region (UTR) and 3’-UTR (Table 1) as described previously [28,29]. Briefly, PCR was carried out using a standard protocol. PCR products were separated electrophoretically in 1.5% agarose gels at ~120 V for 30–45 min in 1x TBE running buffer. Gels were stained with Midori Green and visualized under UV trans-illumination (GENE FLASH) at 302 nm. Product sizes were determined by comparison to the internal 2-log DNA ladder (NEB). The PCR products were purified with a GenElute PCR Clean-Up Kit (Sigma) and ligated into the pGEM-T Easy vector. Stellar Competent Cells (Clontech) were then transformed and plated on MacConkey Agar (Sigma, UK) plates and cultured overnight at 37°C. White colonies were picked and their insert screened by PCR using the vector specific M13 forward and reverse primers. For selected colonies, plasmids were isolated from overnight cultures using a QIAprep Spin Miniprep Kit (Qiagen). Plasmid concentration was determined by Nanodrop analysis and 2 μg of plasmids were sent for Sanger sequencing (Eurofins).

**Table 1:** Primers used for PCR cloning and real-time PCR analysis.

name	5´ to 3´ sequence	application	accession
Trout SelPa2F	CACACTCAGCAGATCGAGCCTG	cloning	MH085053
Trout SelPa2R	GGGTGTTCTAGGTGACCGTAGTCTTCC	cloning	
Salmon SelPa2F	AGCTACTGCTAGACAGAGCTGAGCTGAC	cloning	MH085055
Salmon SelPa2R	TTCTTACATGTTCCACCACCTACACTCC	cloning	
Trout SelPb2F	AGAAATGCAACACACAGCCAGATTACAG	cloning	MH085056
Trout SelPb2R	TACATTTTGGATGCCTAAGTCTACCCTGAC	cloning	
Salmon SelPb2F	ACAGAACACTGCTGGAACAAGAAATGC	cloning	MH085057
Salmon SelPb2R	CGTATCTGACCATGGAACAACCTGG	cloning	
Trout SelPa1F	GCTTGGTGCAGGCATCCTTATTG	qPCR	HF969249
Trout SelPa1R	GGATGGAGTAGGGCAGGGAGATATG	qPCR	
Trout SelPa2_qPCR_F	GGACTGCACGTATGAGAACAC	qPCR	MH085053
Trout SelPa2_qPCR_R	TGCCATGGTGACCGTGCCC	qPCR	
Trout SelPb1-F	GACGACTTCCTGGTATATGACAGATGTG	qPCR	HF969250
Trout SelPb1-R	GGAACTGGGTTGCTGACGGTATC	qPCR	
Trout SelPb2_qPCR_F	CTTTCCTCATTGTGAATGAACG	qPCR	MH085056
Trout SelPb2_qPCR_R	AACTCCATTTGGATTCCTGTCAT	qPCR	
Trout EF1a_F	CAAGGATATCCGTCGTGGCA	qPCR	XM021571866
Trout EF1a_R	ACAGCGAAACGACCAAGAG	qPCR	

### *In vitro* Se supplementation

Two rainbow trout cell lines were used: the fibroblast-like RTG-2 cells [30] and the monocyte/macrophage-like RTS-11 cells [31]. They were grown at 20°C in Leibovitz medium (L-15, Gibco) containing 100 U/ml penicillin and 100 μg/ml streptomycin (P/S), supplemented with 10% foetal bovine serum (FBS, Sigma) for RTG-2 cells and 30% for RTS-11 cells. The cells were passaged at a density of 1x10^6^ cells/ml into 12-well culture plates (Millipore) before stimulation. One day later, the medium was supplemented with either an inorganic (sodium selenite, Na_2_SeO_3_) or an organic (selenocystine L-stereoisomer, L-selenocystine or L-Sec) selenocompound, both purchased from Sigma–Aldrich, at concentrations of 0 nm, 1 nm, 10 nm, 100 nm, 1 μM and 10 μM. The cells were cultured for 24 h and then harvested for RT-qPCR analysis of SelP paralogue expression, as outlined below.

Following on from this preliminary study 100 nM was chosen for a further *in vitro* stimulation experiment. After 24 h with the Se supplements, cells were incubated with stimulants directly dissolved in their culture medium. The following cytokines and pathogen-associated molecular patterns (PAMPs) were used as stimulants: recombinant interferon gamma (IFNγ, 20 ng/ml) [32], interleukin-1β (IL-1β, 25 ng/ml) [33], the bacterial cell wall component lipopolysaccharide (LPS, 25 μg/ml, from *E*. *coli* strain 055:B5, Sigma) and the viral dsRNA mimic polyinosinic: polycytidylic acid (Poly I:C, 50 μg/ml, Sigma), or medium alone as control. The treatments were terminated by dissolving the cells in TRI reagent (Sigma, UK) 6 h post-stimulation and total RNA isolated for cDNA synthesis as described previously [34].

### Fish and ethics statement

Juvenile rainbow trout weighing ~80 g were purchased from College Mill Trout Farm (Perthshire, U.K.) and maintained in 400 L tanks at the University of Aberdeen aquarium facility, supplied with recirculating freshwater at 14°C. All the experiments described comply with the Guidelines of the European Union Council (2010/63/EU) for the use of laboratory animals, and were carried out under UK Home Office project licence PPL 60/4013, approved by the ethics committee at the University of Aberdeen.

### *In vivo* Se supplementation

Fish were acclimated for 2 weeks before starting the feeding trial, and prior PIT tagged for individual identification. They were then fed at 2% body weight/day for 5 weeks prior to use (see section 2.6), with either a control diet containing 0.5 ppm Se (the commercial standard inclusion level) or with an identical diet supplemented with Sel-Plex (Alltech, Nicholasville, KY, USA) at 0.2% inclusion (2g/Kg diet) to achieve 3.51 ppm of mainly organic Se to achieve a supranutritional Se level. The diets were supplied by the Hellenic Center for Marine Research (Athens, Greece). The fish were weighed every two weeks and feed rations updated accordingly. Two replicate tanks per diet were used to reduce variability while assuring sufficient stocking density to avoid pronounced dominance hierarchies from developing amongst the fish.

### Bacterial challenge and sampling

The Hook strain [35] of *Aeromonas salmonicida* ssp. *salmonicida*, a Gram-negative salmonid pathogen, was prepared as described previously [36]. Briefly, the bacteria were grown on a tryptic soy agar plate (TSA, Fluke) for 2 days at 22°C, then scraped off using phosphate-buffered saline (PBS, GIBCO) and washed three times with PBS. The resultant bacteria were re-suspended in PBS containing 15% glycerol and stored at -80°C. The bacterial titre (CFU/ml) was determined by plating on TSA plates in a serial dilution.

For challenge, the *A*. *salmonicida* was injected intraperitoneally (i.p.) into the two groups of rainbow trout (now ~200 g) that were fed with either the control diet or diet supplemented with Sel-Plex, at 2x10^5^ CFU/ml in PBS; 0.5 ml/fish. Fish from both groups were injected with PBS (0.5 ml/fish) alone as uninfected controls. Seven fish from each of the four groups (infected and control) were killed at 48 h post injection and tissues (~100 mg) sampled for gene expression analysis. The tissues were homogenized in 1.5 ml of TRI reagent using a Qiagen Tissue Lyser II, then stored at −80°C until RNA extraction. Total RNA was isolated following the manufacturer’s guidelines. cDNA was synthesized using RevertAid reverse transcriptase (ThermoFisher) in 40 μl reactions, as per the manufacturer’s instructions, then diluted to 600 μl with TE buffer (pH 8.0) and stored at −20°C.

### RT-qPCR analysis

qPCR was performed in a Roche LightCycler 480 using 2× SYBR Green I (Invitrogen) qPCR Master Mix made with a Immolase DNA Polymerase kit (Bioline), with 10 μl reaction mixtures in 384-well plates (Roche) containing 4 μl diluted cDNA in each reaction. The program was set to contain 1 cycle (95°C for 10 min) to denature the cDNA samples and activate the polymerase activity, 40 cycles of amplification (95°C for 30 s, 66°C for 20 s, 72°C for 20 s), followed by melting curve analysis. Program profiles differed for annealing temperature and time for elongation between primer pairs. The annealing temperature for qPCR was 64°C for SelPa1 and SelPb1, 61°C for SelPa2 & SelPb2, 63°C for elongation factor-1α (EF-1α), and that for cloning was 66°C. Data were analysed using LightCycler 480 Software 1.5.1 (Roche). All primers used for RT-qPCR in this study are shown in Table 1 and at least one per pair spans an exon-exon junction to avoid amplification of residual genomic DNA. This was confirmed in separate qPCR reactions with genomic DNA as template. The expression efficiencies and melting curves of each primer pair were assessed for efficiencies above 1.9 and a single peak melting curve using the LightCycler 480 Software 1.5.1 (Roche). The relative expression level of each gene in the respective analysed tissues was absolutely quantified using internal references; serial dilution of equal molar amounts of PCR product from each gene, including the reference gene EF-1α, assessed as optimal for this purpose [37], and individual samples were normalized against their transcript levels of EF-1α.

### Statistical analysis

Relative *in vivo* gene expression values were normalized against EF-1α, scaled and log2 transformed prior to statistical analysis, as described previously [34]. The effects of selenium and/or infection *in vivo* were determined by 2-way ANOVA in the SPSS Statistics package 24 (SPSS Inc., Chicago, Illinois). The *in vitro* expression data was normally distributed, as tested by Levene´s test, and analysed directly by independent student´s t-tests on untransformed expression data. Significance levels were set at p≤0.05.

## Results

### Sequence analysis of salmonid SelPa and SelPb paralogues

The screening of the rainbow trout and Atlantic salmon genome for additional genes encoding selenoproteins yielded further homologues for the selenoproteins Pa and Pb in both species. These newly discovered genes, SelPa2 and SelPb2, were cloned from both rainbow trout and Atlantic salmon cDNA samples and sequenced (**S1–S4 Figs**). An additional trout SelPa2 cDNA sequence (SelPa2-2) was also obtained. It was identical to trout SelPa2 (**S1 Fig**) at the 5’-UTR and coding region but differed at two repeat regions in the 3’-UTR due to tandem repeat polymorphism (**S5 Fig**). The cDNA sequences of the rainbow trout SelPa2, SelPa2-2, SelPb2 and Atlantic salmon SelPa2 and SelPb2 were deposited in Genbank under the accession numbers MH085053, MH085054, MH085056, MH085055 and MH085057, respectively.

**Fig 1:**
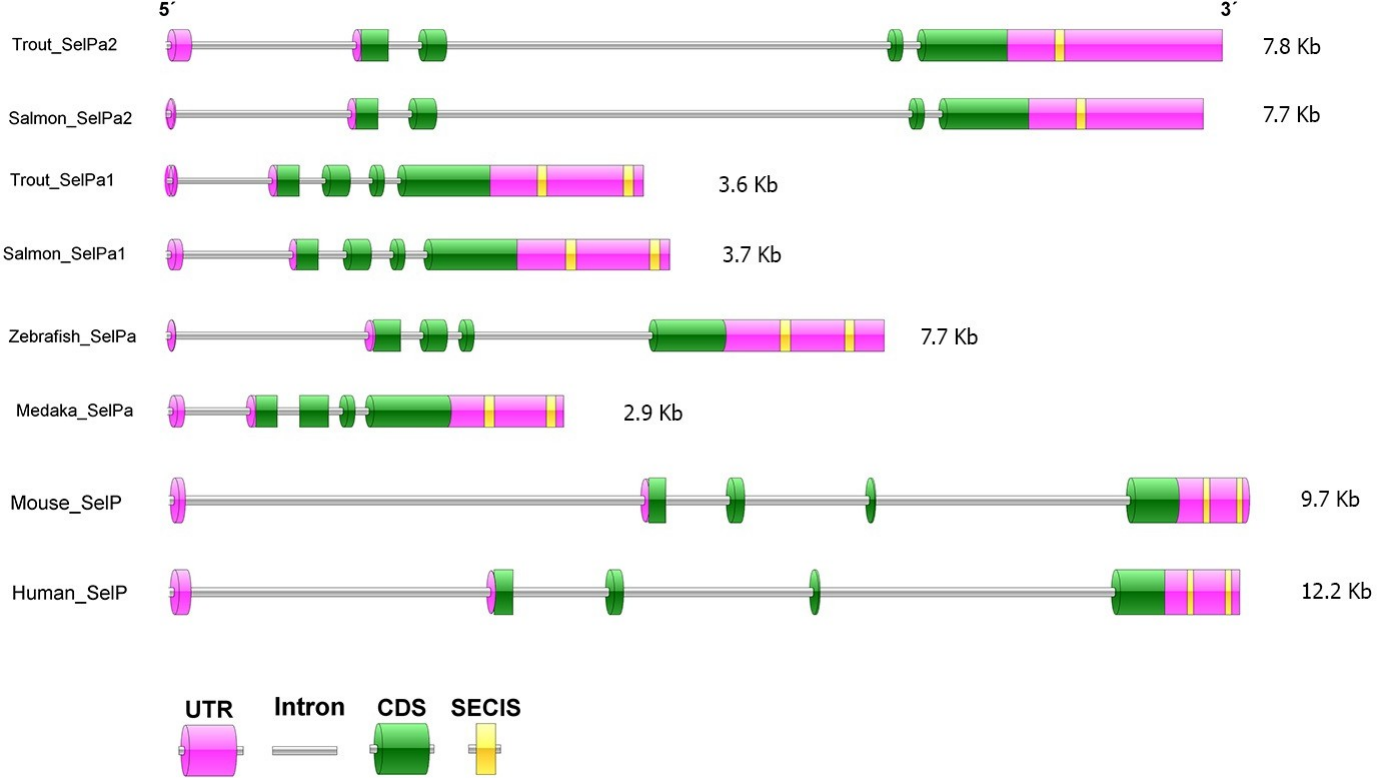
Gene organisation of teleost SelPa in comparison with mammalian SelP. The gene structure was determined by alignments of mRNA with genomic sequences. The exon-intron structure of salmon SelPa paralogues, other fish (zebrafish and medaka) SelPa, and mammalian (mouse and human) SelP are shown. The accession numbers of genomic sequences HF969249 (trout SelPa1), MH085053 (Trout SelPa2), XM_014171135 (salmon SelPa1), MH085055 (salmon SelPa2), NM178297 (zebrafish SelPa), XM_004072219 (medaka SelPa), NM_009155 (mouse SelP), and NM_005410 (human SelP).

**Fig 2:**
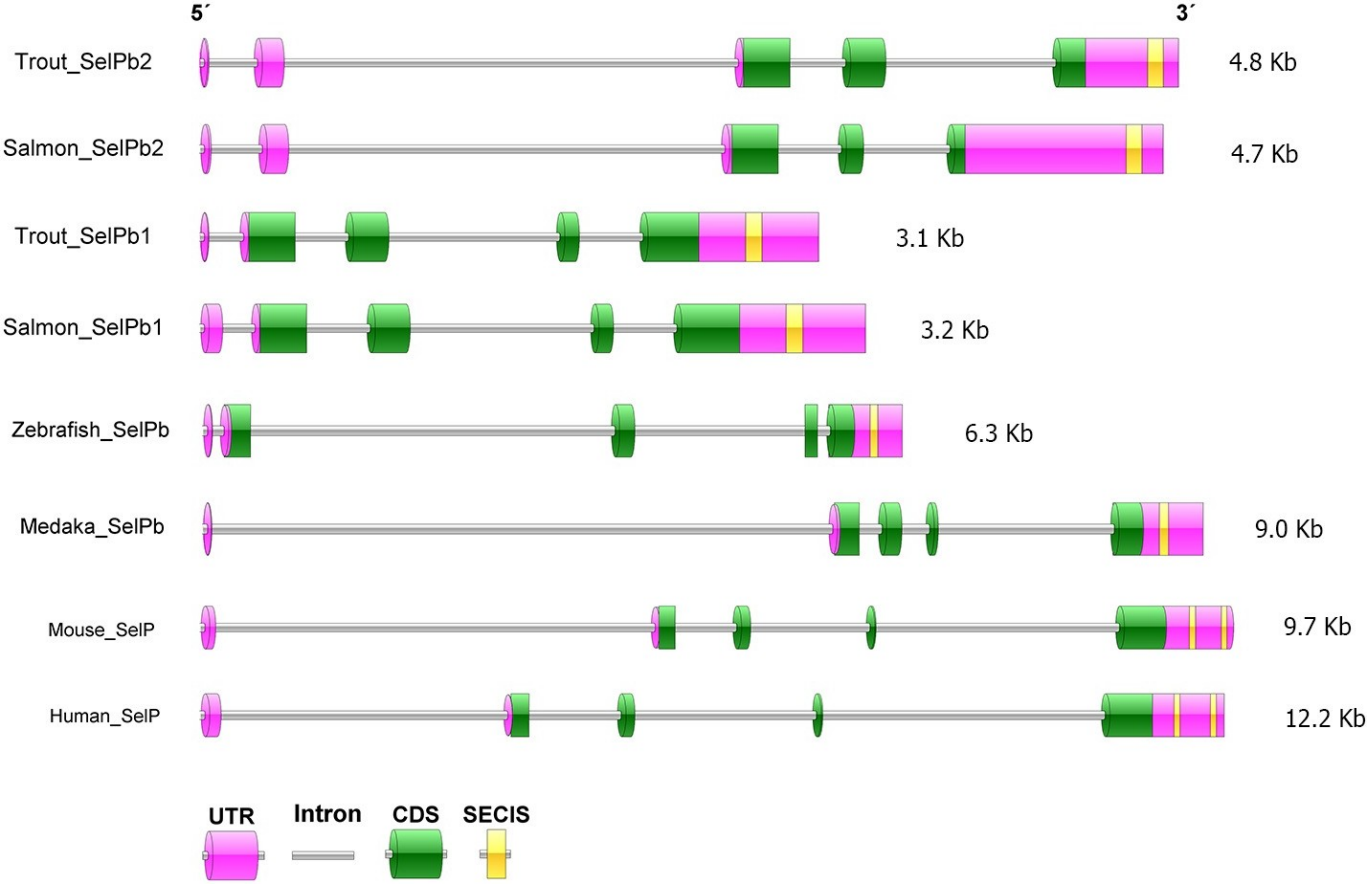
Gene organisation of teleost SelPb in comparison with mammalian SelP. The gene structure was determined by alignments of mRNA with genomic sequences. The exon-intron structure of salmon SelPb paralogues, other fish (zebrafish and medaka) SelPb, and mammalian (mouse and human) SelP are shown. The accession numbers of genomic sequences HF969250 (trout SelPb1), MH085056 (Trout SelPb2), XM_014140446 (salmon SelPb1), MH085057 (salmon SelPb2), NM_001353911 (zebrafish SelPb), XM_004079128 (medaka SelPb), NM_009155 (mouse SelP), and NM_005410 (human SelP).

The exon-intron structure of all discovered SelP genes was predicted with the genomic sequences retrieved from NCBI (**Figs 1 and 2**). Mammalian (human and mouse) SelP and other fish (zebrafish *Danio rerio* and medaka *Oryzias latipes*) SelPa and SelPb genes all have a 5 exon/4 intron structure, with a non-coding first exon. This gene organisation was conserved in trout and salmon SelPa1, SelPa2 and SelPb1 genes but differed in salmonid SelPb2 genes that had the first two exons non-coding despite possessing 5 exons (**Fig 2**). Multiple amino acid (aa) and cDNA alignments suggest independent changes of gene structure of SelPb2 in Atlantic salmon and rainbow trout (described later).

**Fig 3:**
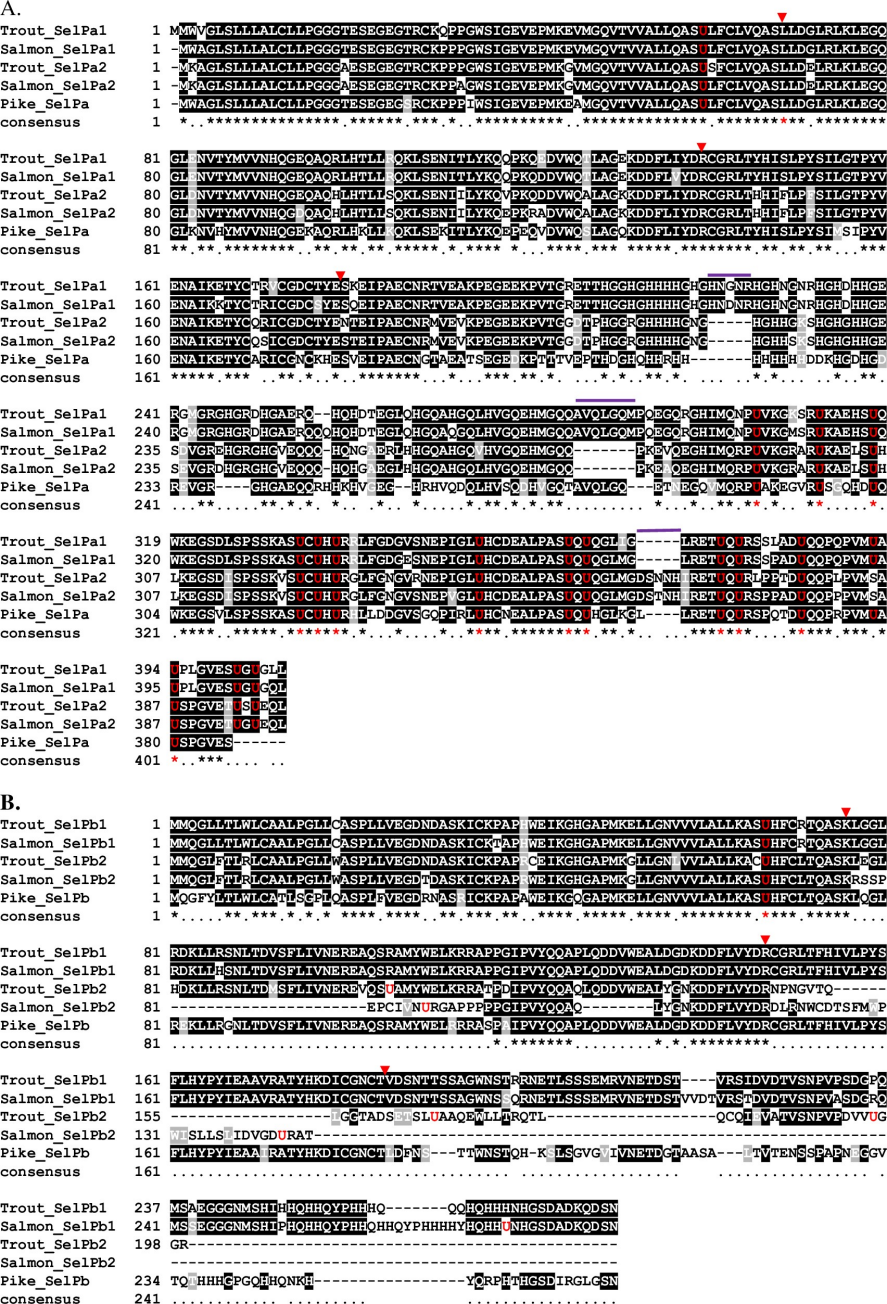
**Amino acid multiple alignments of salmonid SelPa (A) and SelPb (B) together with the pike orthologues.** Sec residues (U) are in red. The intron positions of SelPa and SelPb1 are indicated by red arrowheads. Purple bars over the alignment indicate deletion/insertion of aa blocks. The completely conserved and similar residues are indicated by asterisks and dots (. or :) respectively, below the sequences. The accession numbers of protein sequences analysed are described in S7 Fig.

Each SelP sequence has a complete open reading frame (ORF) confirmed with an in frame stop codon present upstream of the ORF, detected in the cDNA or by alignment with genomic DNA, confirming the translation start codon and therefore complete coding sequence (CDS) is present in the clones. A signal peptide comprising the first 19-20aa was predicted for all deduced proteins (**S1–S4 Figs**). Both trout and salmon SelPa2 cDNAs encode for 399 aa containing 16 Sec residues, compared to 17 Sec in trout and salmon SelPa1. A multiple aa alignment of salmonid SelPa isoforms along with pike SelPa, that may represent an ancestor of salmonid SelP, was produced. The positions of all predicted SEC residues are highly conserved between salmonid SelPa isoforms, as well as pike SelPa (**Fig 3A**). Classically, the N-terminus has a single Sec within an UxxC motif that is encoded by the first coding exon, and the C-terminus contains multiple Sec that are all encoded by the last coding exon. Compared to pike SelPa, blocks of aa sequence deletions / insertions are apparent in an orthologue-specific manner (**Fig 3A**), suggesting these deletions/insertions happened before salmonid speciation.

Whilst trout SelPb2 encodes for a 199aa protein containing 4 Sec residues, salmon SelPb2 encodes for 146 aa containing 3 Sec. A multiple aa alignment of salmonid SelPb and pike SelPb revealed some conservation of salmonid SelPb isoforms, including the N-terminal Sec within an UxxC motif that is again encoded by the first coding exon. The aa sequences encoded by the first two coding exons are well conserved except for a deletion in salmon SelPb2 (**Fig 3B**). However, no similarity was observed thereafter between salmonid SelPb2 and salmonid SelPb1 and pike SelPb (**Fig 3B**). A multiple cDNA alignment of salmonid SelPb paralogues suggests that trout SelPb2 has lost the penultimate exon that led to an ORF shift, and salmon SelPb2 lost the 5’-end of exon 2 (in frame) but incorporated the 3’-portion of intron 2 and retained the intron equivalent to intron 3 of SelPb1 in the coding region that also led to an ORF shift (**S6 Fig**).

The aa identities/similarities between SelP orthologues are generally high, 95.6%/97.5%, 94.5%/96.5% and 91.7%/92.8% for salmonid SelPa1, SelPa2 and SelPb1, respectively. The exception is SelPb2 where a lower identity and similarity is present between the aa sequences (54.8%/57.8%) (**S2 Table**). The aa identities between SelPa paralogues are also high (77.4–78.9%) but are low between salmonid SelPb paralogues (35.9–53.4%), caused by the gene organisation change leading to an ORF shift in SelPb2 (**S6 Fig**).

### Evolutionary analysis of vertebrate SelP

**Fig 4:**
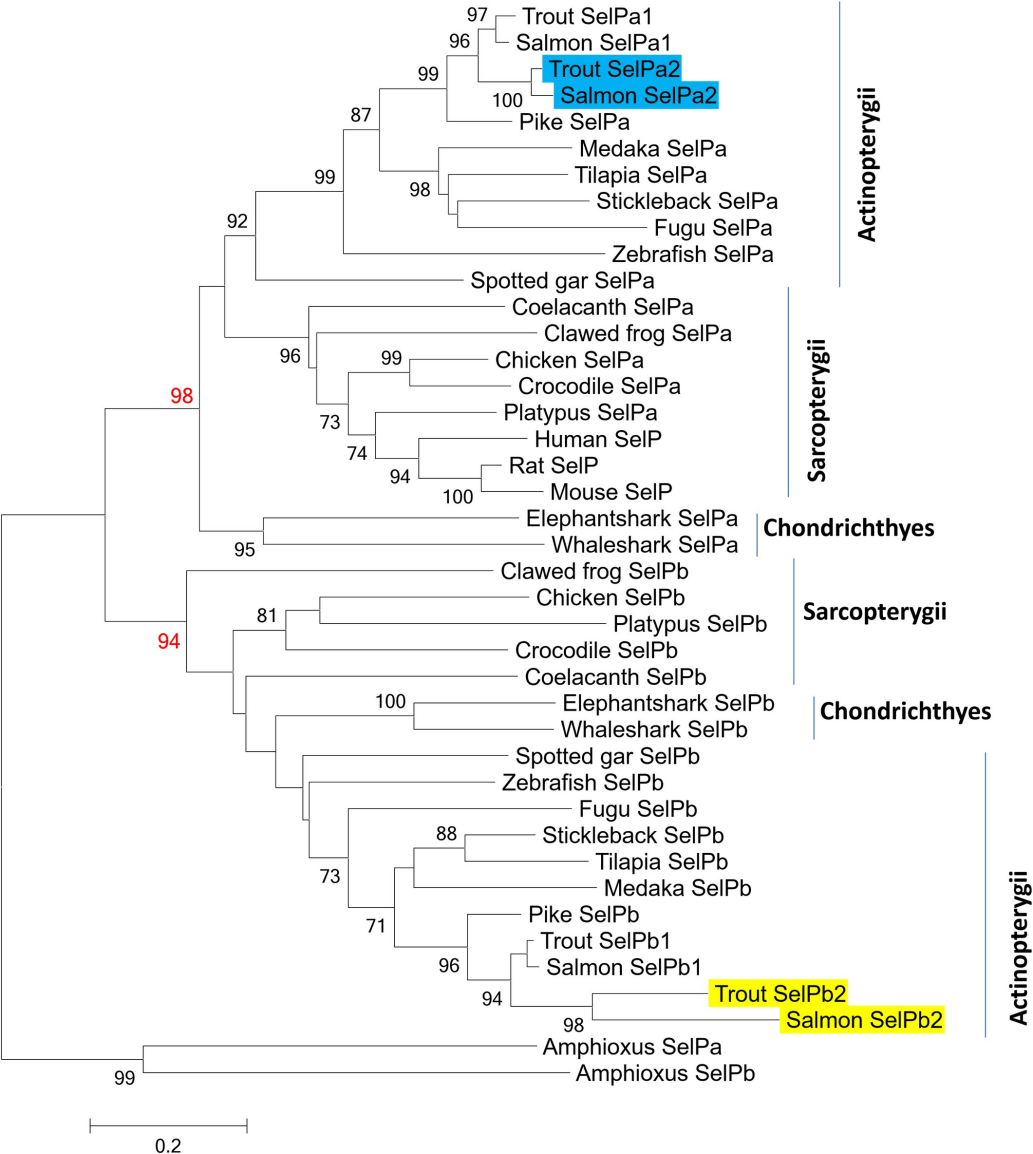
Phylogenetic tree analysis of vertebrate SelPa and SelPb. The phylogenetic tree was constructed using aa multiple alignments (**S7 Fig**) generated by muscle, and the neighbour-joining method using MEGA7 [24]. The invertebrate amphioxus SelP isoforms were used as an outgroup. The evolutionary distances were computed using the JTT matrix-based method with all ambiguous positions removed for each sequence pair. The percentage of replicate trees (when >70%) in which the associated taxa clustered together in the bootstrap test (10,000 replicates) are shown next to the branches. The salmonid SelPa2 and SelPb2 cloned in this study are highlighted in blue and yellow, respectively. The accession number of each sequence is given in S7 Fig.

**Fig 5:**
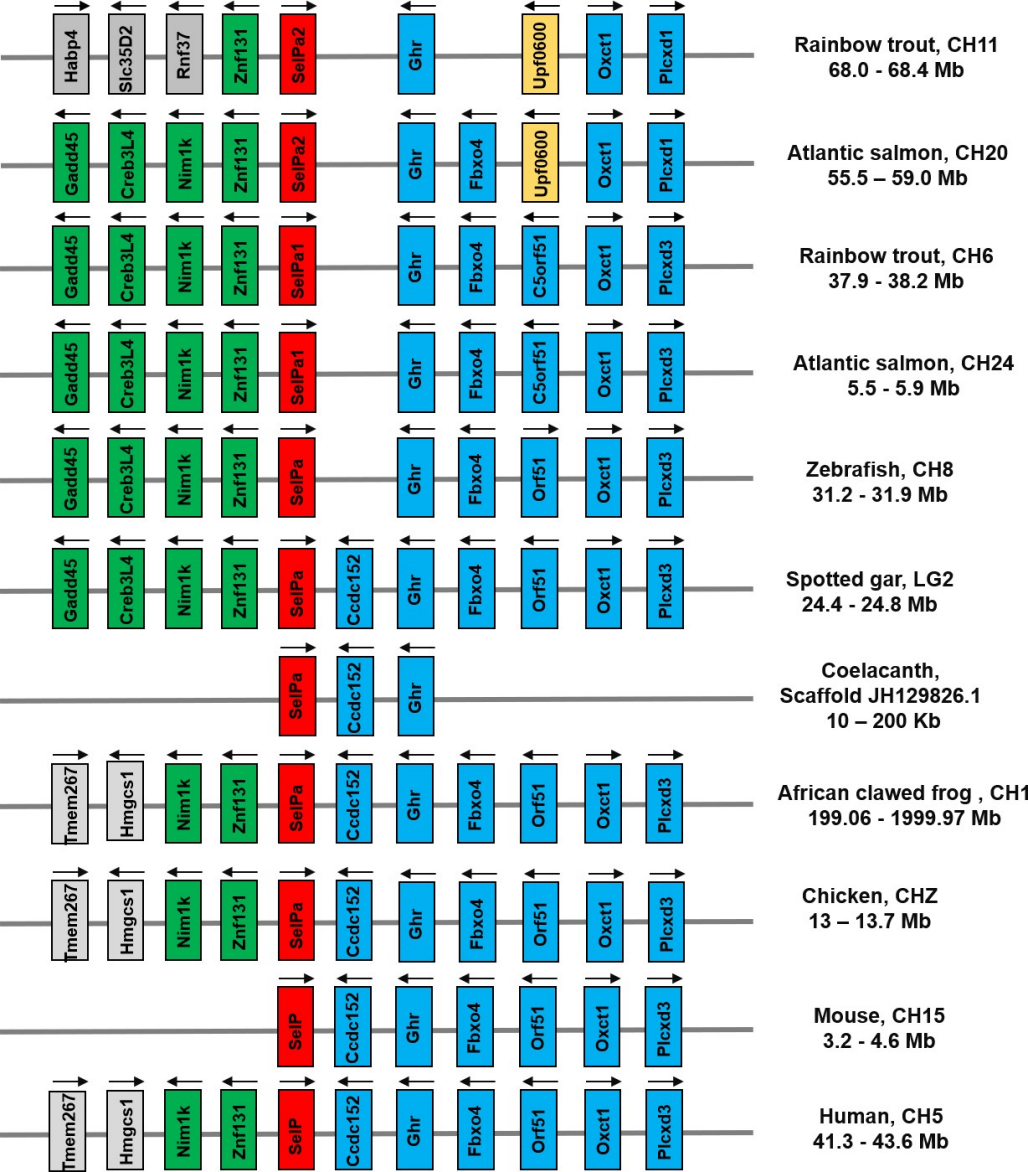
Synteny analysis of vertebrate SelP/SelPa loci. The information on the vertebrate SelPa genomic region was manually extracted from NCBI reference genomes and the ensembl genome browser. SelPa genes are in red. The conserved syntenic genes upstream of vertebrate SelP are in green, and the conserved syntenic genes downstream of vertebrate SelP are in blue. The strand orientation of transcription is indicated by arrows above each gene.

**Fig 6:**
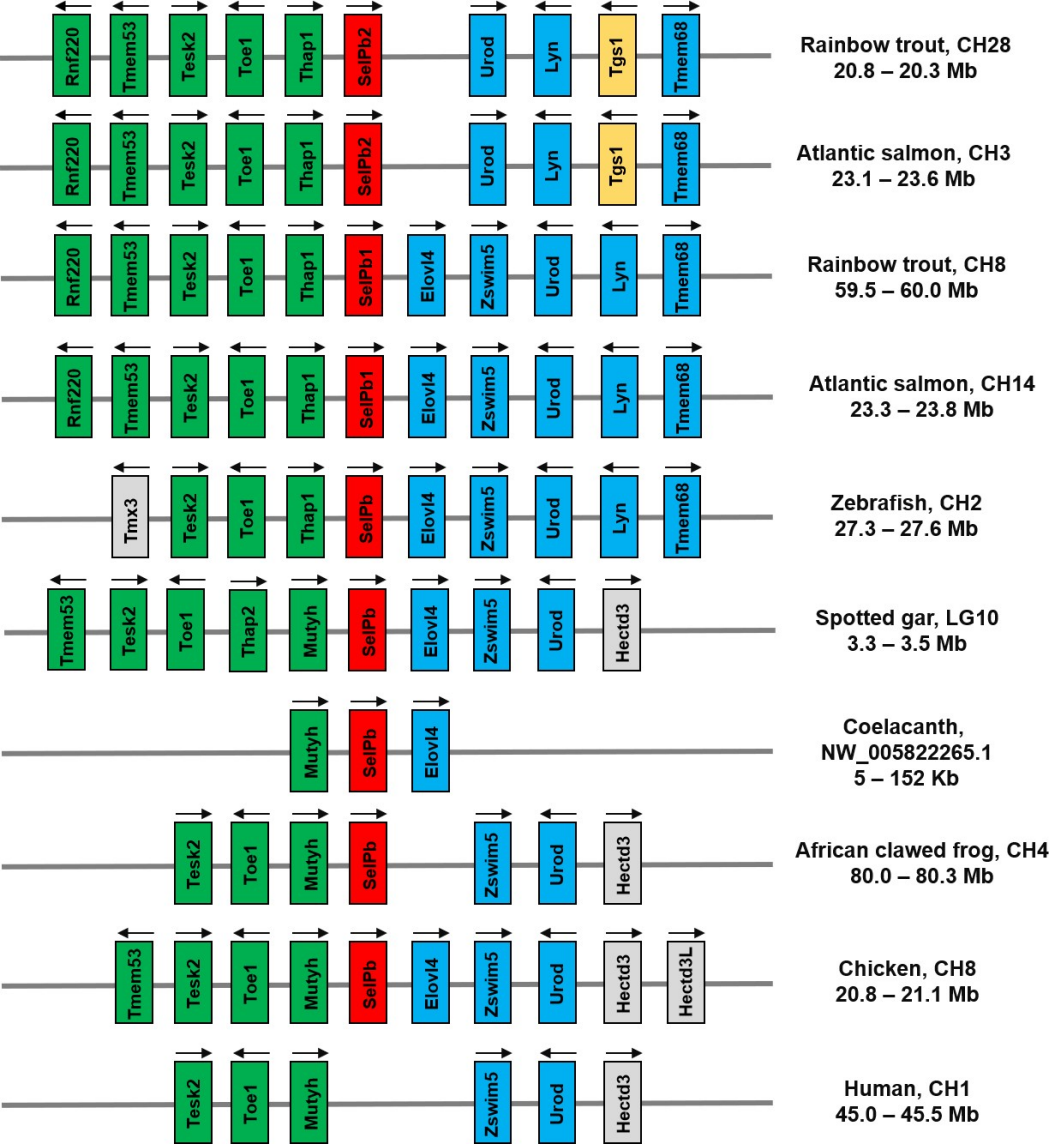
Synteny analysis of vertebrate SelPb loci. The information on vertebrate SelPb genomic region was manually extracted from NCBI reference genomes and the ensembl genome browser. SelPb genes are in red. The conserved syntenic genes upstream of vertebrate SelP are in green, and the conserved syntenic genes downstream of vertebrate SelP are in blue. The strand orientation of transcription is indicated by arrows above each gene.

All the vertebrate SelPa molecules, along with mammalian SelP share high aa identities/similarities within the group compared to those seen between SelPa and SelPb, with SelPb also having high aa identities/similarities within the vertebrates (**S2 Table**), with salmonid SelPb2 proteins showing the least similarity towards the other studied taxa. A neighbour-joining phylogenetic tree (**Fig 4**) using amphioxus SelP molecules as an outgroup verified that two independent SelP clades exist in vertebrates, for SelPa and SelPb. The salmon and trout SelPa1 and SelPa2 form sister clades that group together first, then with the SelPa of their Esociform relative pike before SelPa of other teleost. The identical topology for salmonid taxa was also found in the SelPb clade. This topology represents a classical scenario of paralogues generated from the salmonid 4R WGD [38], suggesting that the salmonid SelPa1/2 and SelPb1/2 arose by this means. This is further confirmed by gene synteny analysis of the SelP loci. Salmonid SelPa1 and SelPa2 are located at different but homologous chromosomes, ie. CH6 and CH11 in rainbow trout and CH20 and CH24 in Atlantic salmon, with neighbouring genes well conserved (**Fig 5**). This conserved synteny was also observed in the SelPa loci in zebrafish, spotted gar, coelacanth, frog and chicken, and SelP in human and mouse. Similarly, salmonid SelPb1 and SelPb2 are located at CH8 and CH28 in rainbow trout, and CH3 and CH14 in Atlantic salmon, with a well conserved synteny that is also observed in the SelPb loci in zebrafish, spotted gar, coelacanth, frog and chicken (**Fig 6**). However, a SelPb gene was missing in this conserved locus on human CH1 (**Fig 6**) and mouse (not shown). Both the phylogenetic tree and synteny analysis support the contention that SelPa and SelPb existed in early Gnathostome vertebrates but that the additional salmonid SelPa and SelPb paralogues arose from the salmonid-specific WGD.

### Tissue-specific expression of SelPa and SelPb paralogues in rainbow trout

**Fig 7:**
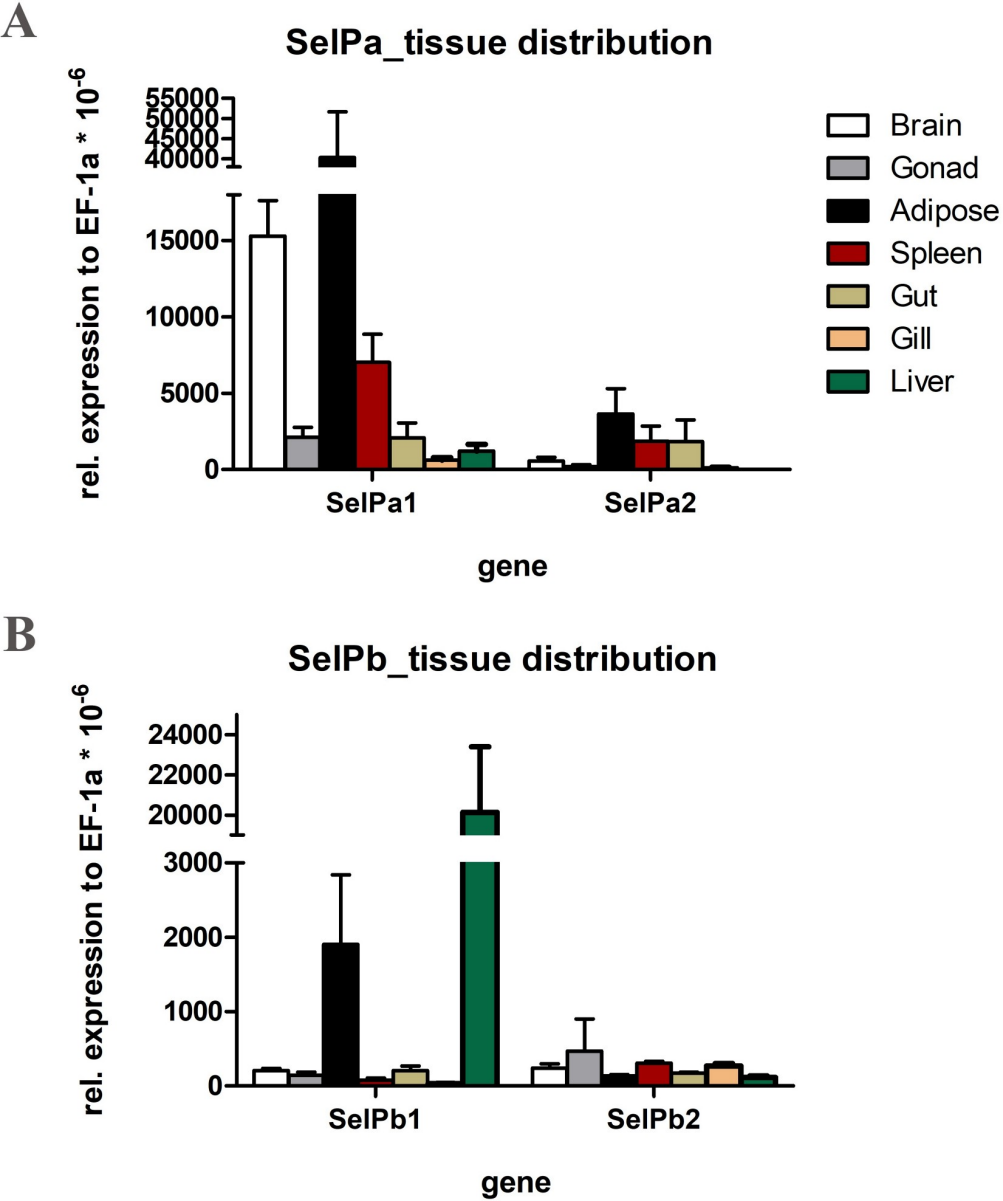
Gene expression of SelPa1/2 and SelPb1/2 paralogue pairs in selected tissues of rainbow trout. The expression of trout SelP genes was quantified using RT-qPCR. The expression of each gene was expressed as arbitrary units relative to that of EF-1a (x1,000,000). Data are means + SEM (N = 5).

Investigation of the tissue specific gene expression of SelP genes in rainbow trout revealed the highest expression of SelPa1 in adipose tissue followed by brain (**Fig *7*A**). SelPa2 also had highest expression in adipose tissue but only a fraction of that of SelPa1. Brain expression of SelPa2 was found to be relatively low. The expression of SelPb1 was highest in the liver followed by adipose tissue (**Fig *7*B)**. SelPb2 showed relatively low overall expression compared to SelPb1. Both SelPa2 and SelPb2 showed no striking differences in tissue specific baseline expression levels.

### Expression and modulation of SelP paralogues *in vitro*

**Fig 8:**
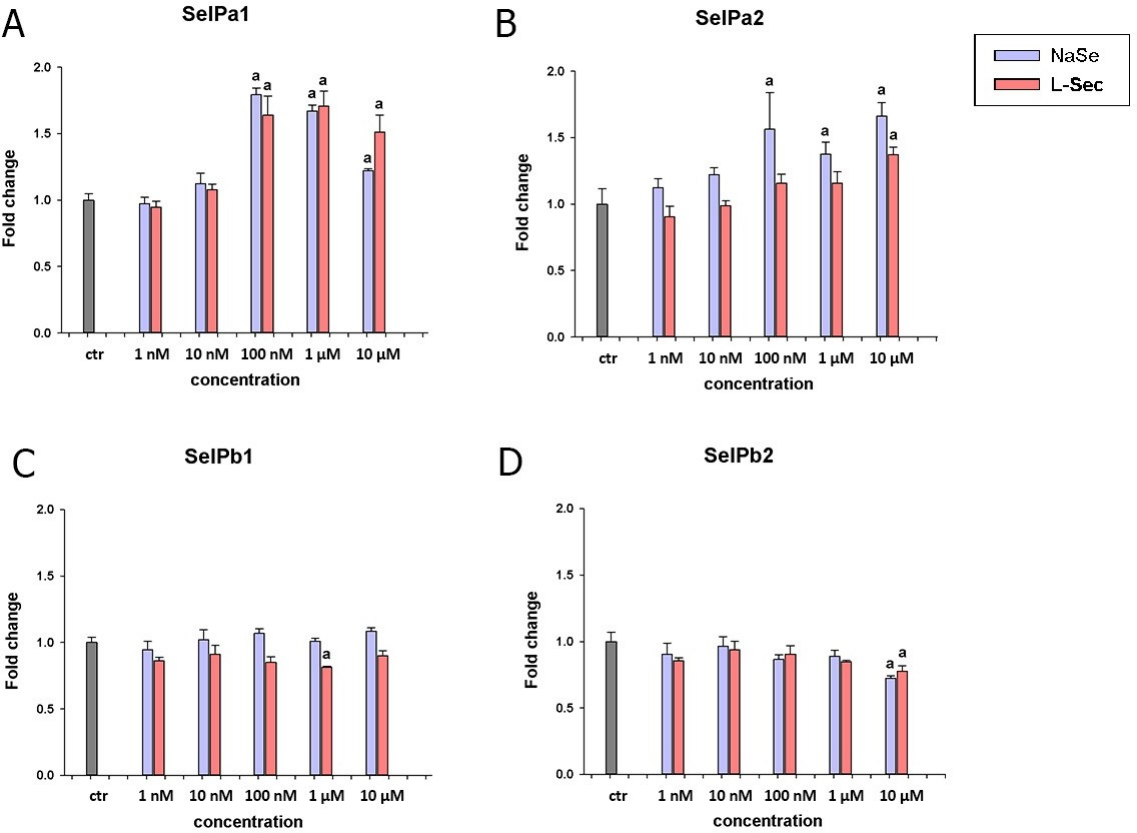
Modulation of SelP transcript expression by seleno-compounds in RTG2 cells. The rainbow trout RTG-2 cell line was incubated for 24 h with the seleno-compounds sodium selenite (NaSe) or L-selenocystine (L-Sec), at concentrations of 0 (control), 1 nM, 10 nM, 100 nM, 1 *μ*M and 10 *μ*M. The expression of SelPa1 (A), SelPa2 (B), SelPb1(C) and SelPb2 (D) was quantified by RT-qPCR and expressed as a fold change. Letters indicate statistically significant differences from the respective unsupplemented controls (ctr). Data are means + SEM of four independent replicates per group.

Expression analysis of the SelP paralogues in the RTG-2 cell line following supplementation with seleno-compounds was next studied (**Fig 8**). Transcripts of SelPa1 were significantly elevated when the cells were exposed to concentrations of 100 nM or higher with sodium selenite or L-selenocystine. Transcript levels of SelPa2 were similarly increased significantly when sodium selenite was supplemented at concentrations of 100 nM or above. However, SelPa2 expression was increased by L-selenocystine only at the highest concentration tested (10 μM). The transcripts of SelPb1 and SelPb2 were not induced by the concentrations of Se tested. In contrast, a small decrease in SelPb1 mRNA level was observed after supplementation with 1 μM L-selenocystine and in SelPb2 after 10 μM of either Se compound.

**Fig 9:**
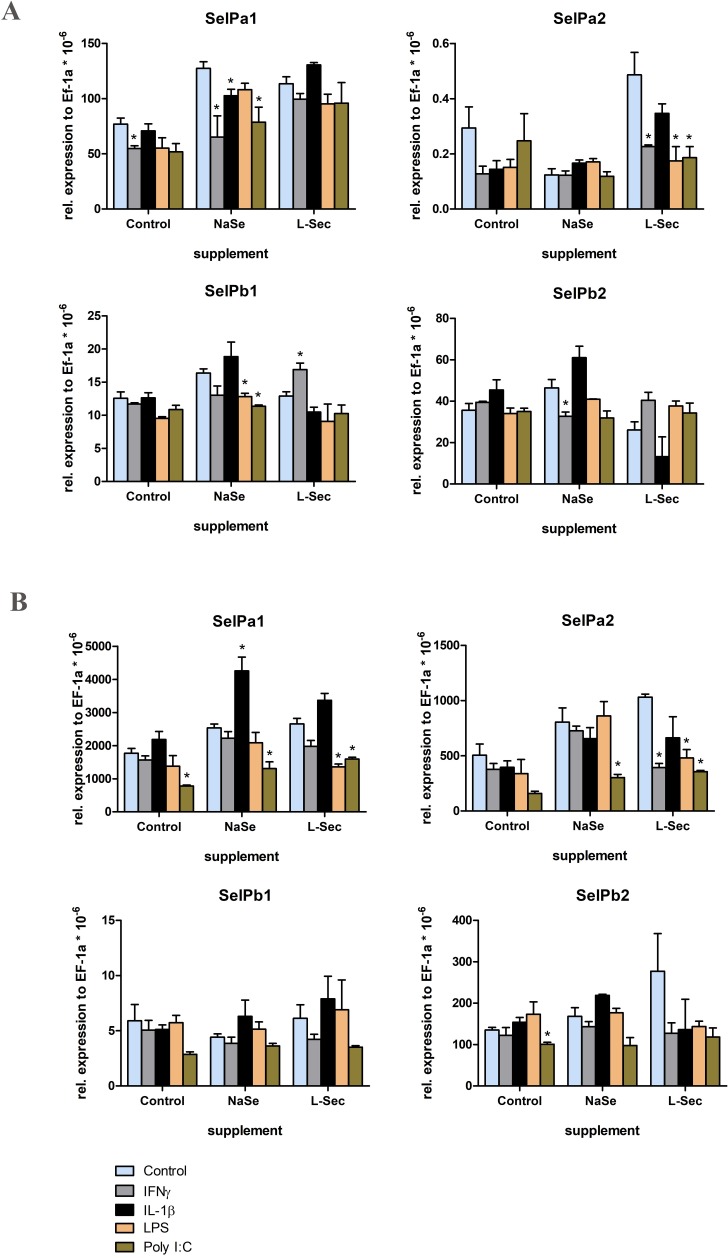
**Effects of selenium supplementation on SelP transcript levels in RTG-2 cells (A) and RTS-11 cells (B) after stimulation with cytokines and PAMPs.** The cells were supplemented with 100 nM sodium selenite (NaSe), 100 nM L-selenocystine (L-Sec) or medium as control for 24 h and then stimulated with IFNγ, IL-1β, LPS and Poly I:C for 6 h. SelP transcript expression was quantified by RT-qPCR as in Fig 7. Data are means + SEM of 3 independent replicates per group. Expression values that are significantly different from the control are marked with an asterisk.

Next RTG-2 and RTS-11 cells were treated with 100 nM seleno-compounds before cytokine and PAMP stimulation, since this dose modulated SelPa expression in RTG-2 cells. The expression of SelP paralogues in control cells was not affected by any of the stimulants tested except IFNγ, that downregulated the expression of SelPa1 in RTG-2 cells, and Poly I:C that downregulated the expression of SelPa1 and SelPb2 in RTS-11 cells (**Fig 9**).

Upon IFNγ stimulation, the expression of SelPa1 and SelPb2 was downregulated in RTG-2 but not RTS-11 cells supplemented with sodium selenite. However, SelPa2 expression was downregulated in both cell lines supplemented with L-selenocystine but not sodium selenite. IL-1β stimulation only affected SelPa1 expression in sodium selenite supplemented cells, with a downregulation seen in RTG-2 cells but an upregulation seen in RTS-11 cells (**Fig 9**).

Only negative effects of seleno-compound supplementation on SelP paralogue expression was observed after PAMP stimulation. LPS downregulated SelPa2 in RTG-2 and RTS-11 cells when supplemented with L-selenocystine, and SelPb1 expression in both cell lines supplemented with sodium selenite (**Fig 9**). Poly I:C downregulated the expression of SelPa1 and SelPb1 in both cell lines when supplemented with sodium selenite, also seen with SelPa1 in RTS-11 cells supplemented with L-selenocystine. SelPa2 expression was also downregulated by Poly I:C when supplemented with L-selenocystine in both cells and in RTS-11 cells with sodium selenite (**Fig 9**).

### Expression and modulation of SelP paralogues *in vivo* by bacterial infection

**Fig 10:**
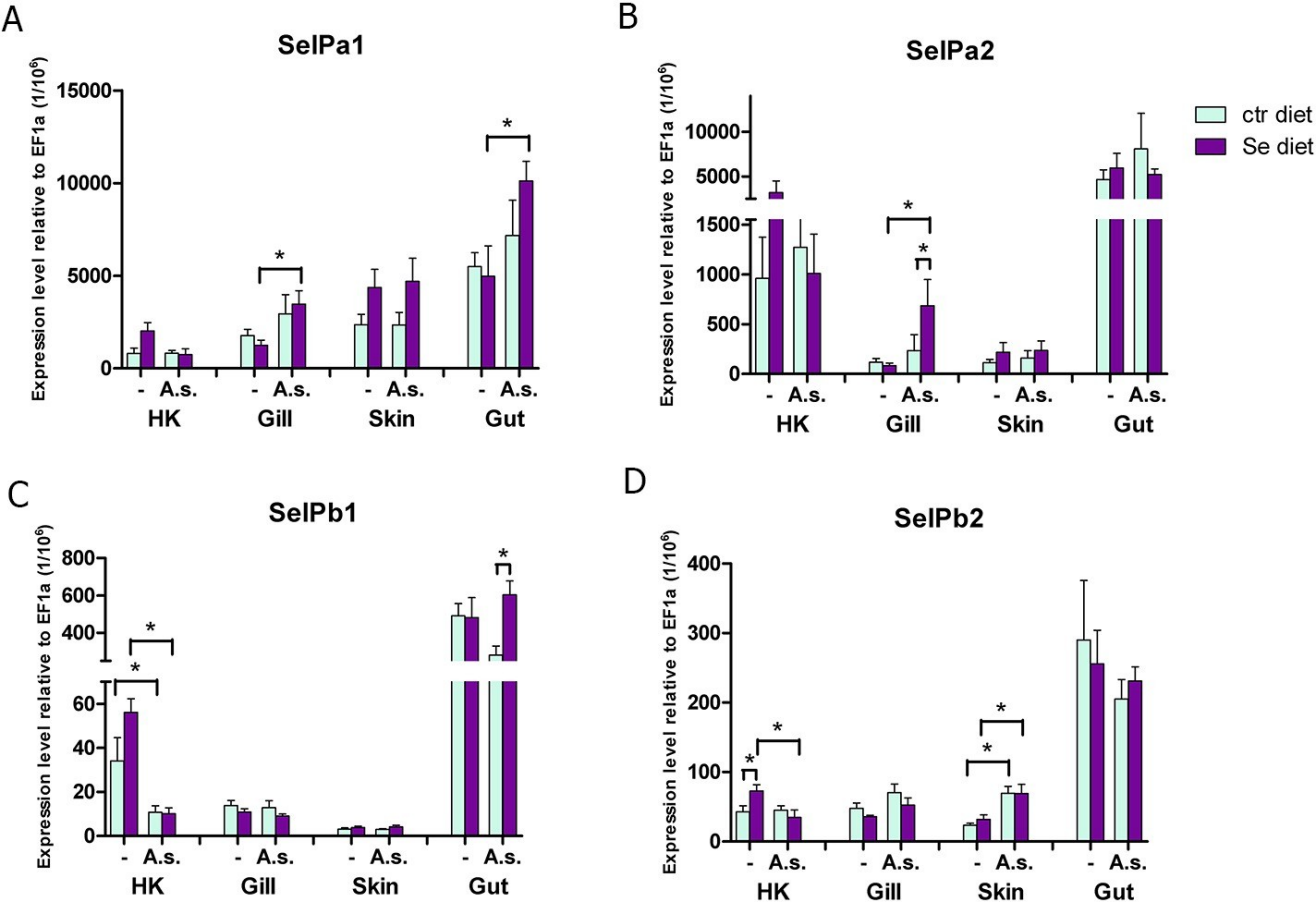
Effects of selenium supplementation on SelP transcript expression after bacterial infection. Rainbow trout were fed with a control diet (ctr diet) or Sel-Plex supplemented diet (Se diet) for 5 weeks prior to ip injection with *A*. *salmonicida* (A.s.) or PBS as control (-). Fish were killed 48 h later and head kidney (HK), gills, skin and gut were collected for gene expression analysis as outlined in Fig 7. The expression of SelPa1 (A), SelPa2 (B), SelPb1 (C) and SelPb2 (D) is presented as means +SEM (N = 7). Expression values are significantly different to the respective unchallenged control fish when marked with an asterisk above a large bracket. Statistically significant differences between diet groups are indicated with an asterisk above a small bracket.

The *in vivo* expression results of the SelP genes in head kidney, gills, skin and gut 48h post infection are shown in **Fig 10**. A significant dietary effect of Se supplementation after 5 weeks of feeding in uninfected fish (PBS controls) was observed only in HK where Se supplementation resulted in a higher transcript level of SelPb2. This trend to higher mRNA levels in the supplemented group was observed in HK for the other SelP paralogues although not statistically significant. After 48 h of infection with the bacterial pathogen *A*. *salmonicida*, SelPa1 was upregulated in gills and gut in fish fed the Se supplemented diet. SelPa2 was also upregulated in response to infection in gills of the Se supplemented group. In HK of infected fish SelPb1 was significantly downregulated independent of dietary group and SelPb2 was downregulated in the Se supplemented group. However, unlike the other paralogues SelPb2 was upregulated in skin in both dietary groups after infection. A diet effect during infection was also observed, for SelPa2 in gills and for SelPb1 in the gut, where the expression after infection was higher in the Se supplemented group.

## Discussion

### Evolution of SelP

The presence of SelPa and SelPb in zebrafish was the first report of a second SelP homologue in any species and led to the discovery of the trout SelPa1 and SelPb1 molecules [16]. When the zebrafish SelP homologues were discovered it was not clear if they arose by tandem gene duplication or whole genome duplication (WGD) [6]. Subsequent studies have confirmed the presence of these two genes in all jawed vertebrate classes [17,18], although absent from most placental mammals, the initial SelP gene duplication supposedly happened early in vertebrate evolution. It is known that SelP-like molecules exist in invertebrates, as seen in amphioxus. In sea lamprey either one [39,40] or two [41] SelP orthologues have been annotated, which we excluded from our phylogenetic analysis due to ambiguity and incompleteness of the sequences. Thus it is unclear whether the SelPa and SelPb paralogues arose before or after the appearance of the vertebrates but the possibility of a WGD event duplicating the locus seems likely from our synteny analysis, with potential loss of one locus (or yet to be found) in lamprey. This is supported by the genomic location of the spotted gar SelP genes reported here, which is in congruence with a reported chromosome-scale genome synteny for a spotted gar-chicken comparison [42]. That paralogs of the 2R gar SelP genes are not apparent in 3R teleosts is peculiar. This leads to the conclusion that the TGD event did not result in two retained paralogues of each ancestral SelP gene in the teleost lineage. In contrast the additional SelPa and SelPb paralogues in salmonids described here seem have arisen by the salmonid specific WGD (Ss4R) event, estimated to have occurred 88 Ma ago [43]. Indeed, the chromosomal regions of ssa24 and ssa20(qa) and of ssa14(qa) and ssa03(p) represent regions in the Atlantic salmon genome that have been assigned to collinear blocks of homology that indicate identifiable sequence duplications [21], as also apparent in our gene synteny analysis of these loci in trout and salmon. These findings confirm the paralogue status of the SelP genes in salmonids.

### Salmonid SelPs

The salmonid SelP paralogues are present as two pairs of SelPa and SelPb. An additional trout SelPa2 cDNA sequence (SelPa2-2) was also obtained. It was identical to trout SelPa2 except for the 3´ UTR, which varied in the copy number of the tandem repeat present and therefore can be considered a tandem repeat polymorphism. SelP variants have also been described in mammals, as seen in rat where four distinct splice variants have been described, that differ in length and Sec content [44]. Some interesting differences in the gene organisation were apparent between the pairs of paralogues. The SelPa2 genes were double the size of SelPa1, primarily due to large size of intron 3. In the case of the SelPb paralogues, the SelPb2 genes were also somewhat larger and had only three coding exons (vs 4 in SelPb1 and the SelPa paralogues). A predicted signal peptide was deduced for all of the SelP proteins in this study in agreement with the secretion of the SelP protein into the blood in mammals. Zebrafish SelPa was found to be secreted when transfected into a mammalian cell line [6,45], but whether this is also the case for teleost SelPb and the new paralogues discovered here needs to be confirmed. Mammalian SelP proteins have been experimentally shown to be glycosylated and in the salmonid sequences at least one potential glycosylation site was present, except for salmon SelPb2 due to the relatively short protein. The number of Sec residues present in the SelPa paralogues was high (16–17) relative to SelPb (1–4). This is associated with the presence of two SECIS sequences in the SelPa1 3’UTR but only one in SelPa2 (and SelPb’s). Efforts to predict a second SECIS in the mRNA extending genomic sequence of SelPa2 returned no results, and raises the question as to whether one SECIS is sufficient for the incorporation of 16 Sec, as predicted in the SelPa2 proteins.

### SelP expression during immune responses *in vitro*

Two rainbow trout cell lines, RTG-2 and RTS-11 cells, were studied for SelP paralogue expression. The relative expression of SelPa1 and SelPb2 exceeded the relative expression levels of SelPa2 and SelPb1 in both cell lines. Also it is interesting to note that in general the expression level of SelPa1 and SelPa2 in RTS-11 (macrophage) cells is much higher than in RTG-2 (fibroblast) cells. This may reflect a higher utilization of these proteins in immunity, perhaps to provide an increased level of protection from the naturally higher levels of reactive oxygen species (ROS) and reactive nitrogen species (RNS) in the RTS-11 cells/macrophages during immune responses.

Following supplementation of the cell culture media with inorganic (sodium selenite) and organic (L-selenocystine) forms of selenium SelPa (but not SelPb) expression was increased, by both Se forms for SelPa1 but only by sodium selenite for SelPa2. This shows that both paralogue sub-functionalisation and Se form specific effects are apparent. Whether bioavailability of the investigated seleno-compounds influences the responses seen cannot be concluded but 24h was considered sufficient for the cells to adjust their cellular Se levels relative to availability in the media. Similarly, whether the *in vitro* Se levels reflect the actual Se status in the cells is unknown but previous studies have shown that ^75^Se from culture media is incorporated into SelP proteins, as seen with zebrafish embryos and HEK-293 cells transfected with zebrafish SelP [46]. SelP expression is inducible by sodium selenite (100 nM, 24 h) *in vitro* in human hepatoma cells, where both mRNA and protein levels increase [47]. In addition, our past studies with a rainbow trout liver-derived cell line (RTL) supplemented with the same Se forms/ dose range used here modulated the expression of another selenoprotein, thioredoxin reductase (trxr)3a, that is known to be an antioxidant and can respond to bacterial infection in head kidney (HK) and spleen of rainbow trout [48]. Whilst clear effects of Se supplementation are apparent in the present study, whether SelP protein production will mirror SelP transcript levels remains to be established.

The *in vitro* Se supplementation was next combined with additional stimulation, namely with cytokines (IFNγ and IL-1β) and PAMPs (LPS and Poly I:C). That SelP expression is influenced by cytokine treatment is known from mammalian studies. For example, SelP expression is downregulated by TNFα stimulation of 3T3-L1 cells [49], by IL-6 in human hepatoma cells [47], and studies of the human SelP promoter have shown it is responsive to IL-1β, IFNγ and TNFα in HepG2 cells [50]. SelPa1 expression in trout RTG-2 cells was significantly downregulated in the sodium selenite treated cells after IFNγ, IL-1β and Poly I:C stimulation, but this was not seen with in cells supplemented with L-selenocystine. The group with no Se supplementation also showed downregulation after IFNγ stimulation, suggesting L-selenocystine was protecting the cells from this effect. In contrast, SelPa2 expression was downregulated by these 3 stimulants but only with L-selenocystine supplementation, again revealing paralogue sub-functionalisation in response to the Se milieu. In RTS-11 cells the SelPa1 and SelPa2 transcripts were consistently downregulated after Poly I:C stimulation, also seen with LPS stimulation after L-selenocystine treatment. However, IL-1β stimulation after sodium selenite treatment upregulated the SelPa1 transcript level in contrast to RTG-2 cells where the same treatment led to reduced SelPa1 expression. This result reveals cell type specific responses are also present. In the case of the SelPb paralogues, the mRNA levels of SelPb1 in RTG-2/RTS-11 cells was lower in the sodium selenite treated cells after stimulation with LPS and Poly I:C, whilst in the L-selenocystine supplemented cells IFNγ stimulation resulted in upregulation of SelPb1. In contrast, SelPb2 transcript levels were downregulated in the sodium selenite group following IFNγ stimulation but no other changes were seen. Overall these gene expression studies of cells in culture show that selenium form and cell type impact selenoprotein expression, as confirmed in previous studies [16,51].

### SelP expression during immune responses *in vivo*

Mammalian SelP is mainly transcribed and translated in the liver before being exported into the plasma and distributed throughout the body [52]. This concept could differ in teleost fish with our finding of highest SelPa1 and SelPa2 mRNA abundance in adipose tissue. Whilst we are not aware of studies of teleost SelP expression in adipose tissue, in 3T3-L1 cells knock down of SelP1 gene expression decreases antioxidant activity, increases inflammation, and impairs adipocyte differentiation [49]. Trout SelPb1 expression is highest in liver but with a relatively high level also in adipose.

Following dietary Se supplementation with Sel-Plex for 5 weeks, only SelPb2 was seen to increase, in the HK, although a trend to higher transcript levels was also seen with the other paralogues in this tissue. In other studies with trout using similar Se inclusion levels (~4 ppm) an increase in SelPa was seen in kidney 6 weeks after feeding [16] and by 10 weeks both SelPa1 and SelPb1 were increased in liver [53]. Perhaps a longer feeding duration would have resulted in significant effects for all genes in this study but nevertheless it is clear that dietary Se supplementation did not affect SelP expression in mucosal tissues (gills, skin, gut).

Following bacterial infection *in vivo*, we found SelPa1 upregulated in gills and gut of infected fish when supplemented with dietary Se for 5 weeks prior to infection. SelPa2 mRNA levels were also found to be higher in gills of the infected fish following Se supplementation. The *in vivo* expression analysis of SelPb showed a downregulation of SelPb1 in HK of the infected fish irrespective of diet, whilst the expression in the gut of infected fish was higher after Se supplementation compared to infected fish without supplementation. The transcripts of SelPb2 were also lower in HK of infected fish compared to uninfected fish in the Se supplemented group, whilst transcripts in skin were increased in infected fish from both groups. It is very interesting that changes in SelP expression are apparent 48h post infection, when the immune response is still in the innate stage but already initialising an adaptive response. The role of SelP in infection is not well understood. It has been described as a negative acute phase protein in mammals, with data suggesting a repression during the acute phase response [50]. In salmon SelPa1 is downregulated in macrophages and HK after infection with the intracellular bacterium *Piscirickettsia salmonis* [54], whilst in trout downregulation of SelPa1 was seen in HK and SelPb1 in liver after Poly I:C injection, used as mimic of viral infection [53]. Downregulation of SelP in infection/acute phase reaction may represent a mechanism to generate Se for incorporation into other selenoproteins that are more directly involved in immunity e.g. GPx1, TrxR1 and Selenoprotein K [55,56]. Indeed, we have previously observed an enhanced expression of GPx1, TrxR, Fep15 and Msrb1 in HK of infected fish (unpublished). However, that some paralogues were induced in mucosal tissues in this study could reflect the role of SelP as a selenium transport protein or its peroxidase function mediated by the N-terminal domain or both, with differential tissue responses enabled by the presence of multiple paralogues.

## Conclusions

Two new SelP genes, namely SelPa2 and SelPb2, have been identified in rainbow trout and Atlantic salmon in the present study. Despite 16 predicted Sec residues in the SelPa2 genes only a single SECIS element could be predicted. The SelPb2 protein is truncated but has a higher content of Sec compared to SelPb1. The *in vitro* expression study showed that both paralogues of SelPa are highly expressed in RTS-11 cells compared to RTG-2 cells and that supplementation of organic and inorganic selenium impacts SelPa and SelPb expression levels upon stimulation. The *in vivo* study further demonstrated an interaction of dietary Se and infection on SelP expression in different tissues, in an isoform specific manner. The multiple Sec residues in SelP proteins (especially SelPa1 and SelPa2) are a unique characteristic of this selenoprotein and could indicate an enhanced requirement for selenium in this fish lineage. Hence the optimal dietary inclusion level of Se for salmonid aquaculture might need to be re-evaluated.

## Supporting information

S1 Fig Nucleotide and deduced amino acid sequences of rainbow trout SelPa2. (DOCX)

S2 Fig Nucleotide and deduced amino acid sequences of Atlantic salmon SelPa2. (DOCX)

S3 Fig Nucleotide and deduced amino acid sequences of rainbow trout SelPb2. (DOCX)

S4 Fig Nucleotide and deduced amino acid sequences of Atlantic salmon SelPb2. (DOCX)

S5 Fig Alignment of the two trout SelPa2 cDNA sequences cloned in this study. (DOCX)

S6 Fig cDNA sequence alignment of salmonid SelPb paralogues (A) and schematic gene organisation of the coding region (B).
(DOCX)

S7 Fig Protein alignment of all selenoprotein P sequences used in the phylogenetic analysis. (DOCX)

S1 Table Predicted SECIS sequences and 2D structure for trout and salmon SelP genes as predicted from their mRNA sequences. (DOCX)

S2 Table Amino acid sequence identity (top right) and similarity (bottom left) of vertebrate SelPa and SelPb. The accession numbers are as in S7 Fig.
(DOCX)
