# Supplementary material for: Four selenoprotein P genes exist in salmonids: Analysis of their origin and expression following Se supplementation and bacterial infection
Source: PLoS One. 2018 Dec 20;13(12):e0209381. doi: 10.1371/journal.pone.0209381 (PMC6301783; doi:10.1371/journal.pone.0209381)
Supplement: S1 Fig — (DOCX) [file pone.0209381.s001.docx]

**S1 Fig**

| 1 76 151 226 1 301 23 376 48 451 73 526 98 601 123 676 148 751 173 826 198 901 223 976 248 1051 273 1126 298 1201 323 1276 348 1351 373 1426 398 1501 1576 1651 1726 1801 1876 1951 2026 2101 2176 2251 2326 2401 2476 2551 2626 2701 2776 2851 2926 3001 | TCACACACAGATGTG CAGAAGTGCATATGC ACAGAACCCCATCAT GAACTTTGTCTTCCT TTCGGGTATACAGGA AAATAACAGTGCGAG TATAAAAGGGGGGAG CAGAGGGAGTTGTTG TTGTTCCAAAAGCTA CTGCTAGACAGAGCT GAGCTGACTAAGGAG GGACATTTTAACAGA GGTCTCTTCTACTCA CACACACTCAGCAGA TCGAGCCTGCTGCAA GGCACCGTGATGAAG GCGGGGCTCAGCCTG CTCCTGGCTCTCTGC CTGCTCCCTGGGGGC GGAGCAGAGAGTGAG   M  K   A  G  L  S  L   L  L  A  L  C   L  L  P  G  G   G  A  E  S  E  GGGGAGGGGACCCGC TGTAAGCCACCACCT GGTTGGAGCATTGGG GAGGTGGAGCCAATG AAGGGGGTTATGGGC  G  E  G  T  R   C  K  P  P  P   G  W  S  I  G   E  V  E  P  M   K  G  V  M  G  CAGGTCACGGTGGTG GCCCTCCTCCAGGCC AGCTGATCGTTCTGC TTGGTGCAGGCATCC TTATTGGATGAGCTG  Q  V  T  V  V   A  L  L  Q  A   S  U  S  F  C   L  V  Q  A  S   L  L  D  E  L  CGCCTGAAGCTGGAG GGCCAGGGTCTGGAC AATGTGACCTATATG GTGGTGAACCACCAG GGGGAGCAGGCCCAG  R  L  K  L  E   G  Q  G  L  D   N  V  T  Y  M   V  V  N  H  Q   G  E  Q  A  Q  CACCTTCACACCTTG CTGAGCCAGAAACTG TCTGAGAACATCATA TTGTACAAACAGGTA CCCAAACAGGATGAC  H  L  H  T  L   L  S  Q  K  L   S  E  N  I  I   L  Y  K  Q  V   P  K  Q  D  D  GTGTGGCAGGCCCTG GCTGGAAAGAAGGAT GACTTCCTCATCTAT GACAGGTGTGGTCGT CTGACCCACCATATC  V  W  Q  A  L   A  G  K  K  D   D  F  L  I  Y   D  R  C  G  R   L  T  H  H  I  TTCCTCCCCTTTTCC ATCCTGGGTACTCCC TACGTAGAGAACGCC ATTAAGGAGACCTAC TGCCAACGCATCTGT  F  L  P  F  S   I  L  G  T  P   Y  V  E  N  A   I  K  E  T  Y   C  Q  R  I  C  GGGGACTGCACGTAT GAGAACACAGAGATC CCAGCAGAGTGCAAC AGGATGGTAGAGGTA AAGCCTGAGGGAGAA  G  D  C  T  Y   E  N  T  E  I   P  A  E  C  N   R  M  V  E  V   K  P  E  G  E  GAGAAGCCAGTCACT GGAGGGGATACACCT CACGGTGGACGCGGC CATCATCACCATGGC AATGGGCACGGTCAC  E  K  P  V  T   G  G  D  T  P   H  G  G  R  G   H  H  H  H  G   N  G  H  G  H  CATGGCAAAAGCCAT GGTCACGGTCACCAT GGGGAGAGTGATGTG GGGCGCGAACACGGT CGTGGCCATGGGGTG  H  G  K  S  H   G  H  G  H  H   G  E  S  D  V   G  R  E  H  G   R  G  H  G  V  GAGCAGCAGCAGCAC CAAAATGGCGCTGAG AGGCTCCACCATGGC CAGGCCCATGGCCAA GTGCACGTTGGTCAG  E  Q  Q  Q  H   Q  N  G  A  E   R  L  H  H  G   Q  A  H  G  Q   V  H  V  G  Q  GAGCATATGGGTCAG CAGCCCAAGGAGGTG CAGGAAGGGCATATT ATGCAGAGGCCCTGA GTGAAGGGGAGGGCC  E  H  M  G  Q   Q  P  K  E  V   Q  E  G  H  I   M  Q  R  P  U   V  K  G  R  A  AGGTGAAAGGCAGAG CTCAGCTGACATTTG AAGGAGGGGTCTGAC ATAAGTCCCTCCTCC AAGGTCAGCTGATGC  R  U  K  A  E   L  S  U  H  L   K  E  G  S  D   I  S  P  S  S   K  V  S  U  C  TGACACTGACGGGGG CTGTTTGGCAATGGG GTGCGCAACGAGCCA ATCGGGCTCTGACAC TGTGATGAGGCGCTG  U  H  U  R  G   L  F  G  N  G   V  R  N  E  P   I  G  L  U  H   C  D  E  A  L  CCCGCCTCCTGACAG TGACAGGGACTGATG GGCGACTCCAACAAT CACATCAGGGAGACC TGACAGTGACGCTTG  P  A  S  U  Q   U  Q  G  L  M   G  D  S  N  N   H  I  R  E  T   U  Q  U  R  L  CCCCCCACAGACTGA CAGCAGCCTCTGCCA GTGATGTCAGCCTGA TCCCCGGGTGTTGAA ACCTGAAGCTGAGAG  P  P  T  D  U   Q  Q  P  L  P   V  M  S  A  U   S  P  G  V  E   T  U  S  U  E  CAGCTGTAAGCAGGG CCATGGCTCTGTGAA AGTTATCTTGCTTAT AGGCCTCATATTAAC ATCTGTGAGACCAAC  Q  L  *    ACCGGGGCAAAGCGC TCATACCATCTTACC AACTAGTATTGCTCC GGTACTGTAAAGCCA CACTTGATAGAGAGG GATGGAGGAATACAT TGGGTCTGGTAGAAG AATGCTAAGCCTAAA CCTGTTACGATATGG GGAATAGGATAGTAT TTGAGATGTACCCAA AGGGATAGTATGACA TCAAGGAAGGTAGCC TCCACCCTCTCTTTC TCTGTCGGTGCATTG GTGTCACCATCCCGG GAGTGTATTGGGTGA CTATTTTGATGTTTC TCCCCCTCCGTTTTG CTCCCTCCATCCAAA ATGAAGGTAGGCACA GAAACTACGCTGTAG TGGTGTCTGTCTGAT GTCCGGCTGGGGAGA GGGGAGGGTGAGGGC CATACAATATGACCA CCCCCTTAATTCAAA GTTCACAATATCGAG AGCTTTAGAAGGAGT ATGAAGGAGTAGCGT CCAATGTTGAAATAA ACTGAAATCATGGCC AGAAATGCAAGATTT GAACCAAAGTTAGAC ATTGTTATATTCAGC GTTAGTTAGACATTG TTATACTCAGCGTTA GTTAGACATTGTTAC ATTCAGCGTTAGTTA GACATTGTTATACTC AGCGTTAGTTAGACA TTGTTATACTCAGCG TTAGTTAGACATTGT TATACTCAGCGTTAG TTAGACATTGTTATA CTCAGCGTTAGTTAG ACATTGTTATATTCA GCGTTAGTTAGACAT TGTTATACTCAGCGT TAGTTAGACATTGTT ATACTCAGCGTTAGT TAGACATTGTTATAT TCAGCGTTAGTTAGA CATTGTTATACTCAG CGTTAGTTAGACATT GTTATATTCAGCGTT AGTTAGACATTGTTA TACTCAGCGTTAGTT AGACATTGTTATACT CAGCGTTAGTTAGAC ATTGTTATATTCAGC GTTAGTTAGACATTG TTATATTCAGCGTTA GTTAAACATTGTTAT ATTCAGTGTTAGTTA GATATTGTTATATTC AGCGTTAGTTAAACA TTGTTATATTCAGTG TTAACAAAACACATT TGAATAAAATACATT ATTGATAGAGTAAGA TTTTTCTATTATGAC CCCATCAACCTTCTC AACTTTTTCTTCAAC TAAAAATCTAATGTC ACTCCTTTCAAGCAA CAACATTTATTTGTA CCTTTGAATTAACGC TGTGTTTATTAAAGC AGTAGTGTGTTTTGT AGACAAAATTCAATA TTCATAGTGTACAGC AGTGATAATGTGTGT TTAGTTTTCCATGTT TCTGTCACTGTTATT GCTGTTGGCAATATT ACACATATCTATAAT GCACTGTTCATGAAC TAACAAAACAAAGTG ACCACTTTTCAACTA ACAAAATATATTACA CACATATCTATAATG CACTGTTCATGAACT AACAAAACAAAGTGA CCGCTTTTCAACCAA CAAAAAAACAGCTGG AATGAAGGTGGGTAG AGAGAAATATTGAAT ATACCGTACTTAATG AAGGTCTCTGGGGAA GACTACGGTCACCTA GAACACCC |
| --- | --- |

**S1 Figure: Nucleotide and deduced amino acid sequences of rainbow trout SelPa2.** The cDNA sequence was obtained by PCR. The start and stop codons for the main open reading frame (ORF) and the immediate upstream stop codon of the main ORF are highlighted in red. The TGA codon for Sec (U) is highlighted in green. The primer binding sites for PCR amplification are boxed. Intron positions are indicated by red arrowheads. A predicted signal peptide is highlighted in green. The predicted SECIS element is highlighted in yellow and boxed. A region of predicted tandem repeats (23 copies of 26 bp repeats with a consensus sequence of AGTTAGACATTGTTATATTCAGCGTT) is shaded in grey. The nucleotide sequence in blue is derived from an EST.
