# Supplementary material for: Four selenoprotein P genes exist in salmonids: Analysis of their origin and expression following Se supplementation and bacterial infection
Source: PLoS One. 2018 Dec 20;13(12):e0209381. doi: 10.1371/journal.pone.0209381 (PMC6301783; doi:10.1371/journal.pone.0209381)
Supplement: S2 Fig — (DOCX) [file pone.0209381.s002.docx]

S2 Figure

| 1  78 1 153 25 228 50 303 75 378 100 453 125 528 150 603 275 678 200 753 225 828 250 903 275 978 300 1053 325 1128 350 1203 375 1278 400 1353 1428 1503 1578 1653 1728 1803 1878 1953 2028 2103 2178 2253 2328 2403 2478 2553 | AGCTACTGCTAGACAGAGCTGAGCTGACTAA GGAGGGAGATTTTAACAGAGGCAGATCGAGC CTGCTGCACGGCACC GTGATGAAGGCGGGG CTCAGCCTGCTCCTG GCTCTCTGCCTGCTC CCTGGGGGCGGAGCA GAGAGTGAGGGGGAG    M  K  A  G   L  S  L  L  L   A  L  C  L  L   P  G  G  G  A   E  S  E  G  E  GGGACCCGCTGTAAG CCACCAGCTGGTTGG AGCATTGGGGAGGTG GAGCCAATGAAGGGG GTTATGGGCCAGGTC  G  T  R  C  K   P  P  A  G  W   S  I  G  E  V   E  P  M  K  G   V  M  G  Q  V  ACGGTGGTGGCCCTC CTCCAGGCCAGCTGA TTGTTCTGCTTGGTGCAGGCATCCTTATTG GATGAGCTGCGCCTG  T  V  V  A  L   L  Q  A  S  U   L  F  C  L  V  Q  A  S  L  L   D  E  L  R  L  AAGCTGGAGGGCCAG GGTCTGGACAATGTG ACCTATATGGTGGTG AACCACCAGGGGGAC CAGGCCCAGCACCTT  K  L  E  G  Q   G  L  D  N  V   T  Y  M  V  V   N  H  Q  G  D   Q  A  Q  H  L  CACACCTTGCTGAGC CAGAAACTGTCTGAG AACATCATATTGTAC AAACAGGAGCCCAAA CAGGCTGACGTGTGG  H  T  L  L  S   Q  K  L  S  E   N  I  I  L  Y   K  Q  E  P  K   Q  A  D  V  W  CAGGCCCTGGCTGGA AAGAAGGATGACTTC CTCATCTATGACAGG TGTGGTCGTCTGACC CATCATATCTTCCTC  Q  A  L  A  G   K  K  D  D  F   L  I  Y  D  R   C  G  R  L  T   H  H  I  F  L  CCTTTTTCCATCCTG GGTACTCCCTACGTA GAGGACGCCATTAAG GAGACCTACTGCCAA AGCATCTGTGGGGAC  P  F  S  I  L   G  T  P  Y  V   E  D  A  I  K   E  T  Y  C  Q   S  I  C  G  D  TGCACGTATGAGAGC ACAGAGATCCCAGCA GAGTGCAGCAGGATG GTAGAGGTAAAGCCT GAGGGAGAAGAAAAG  C  T  Y  E  S   T  E  I  P  A   E  C  S  R  M   V  E  V  K  P   E  G  E  E  K  CCAGTTACTGGAGGG GATACACCTCACGGT GGGCGCGGCCATCAT CACCATGGCAATGGG CACGGTCCCCATAGC  P  V  T  G  G   D  T  P  H  G   G  R  G  H  H   H  H  G  N  G   H  G  P  H  S  AAAAGCCATGGTCAC GGTCACCATGGCGAG AGTGAGGTGGGGCGC GATCACGGTCGTGGC CATGGGGTGGAGCAG  K  S  H  G  H   G  H  H  G  E   S  E  V  G  R   D  H  G  R  G   H  G  V  E  Q  CAGCAGCACCAACAT GGCGCTGAGGGGCTC CACCATGGCCAGGCC CATGGCCAATTGCAC GTTGGTCAGGAGCAT  Q  Q  H  Q  H   G  A  E  G  L   H  H  G  Q  A   H  G  Q  L  H   V  G  Q  E  H  ATGGGTCAGCAGCCC AAGGAGGCGCAGGAA GGGCATATTATGCCG AGGCCCTGAGTGAAG GGGAGGGCCAGGTGA  M  G  Q  Q  P   K  E  A  Q  E   G  H  I  M  P   R  P  U  V  K   G  R  A  R  U  AAGGCGGAGCTCAGC TGACATTTGAAGGAG GGGTCTGATATAAGT CCCTCCTCCAAGGTC AGCTGATGCTGACAC  K  A  E  L  S   U  H  L  K  E   G  S  D  I  S   P  S  S  K  V   S  U  C  U  H  TGACGGGGGCTGTTT GGCAATGGGGTGAGC AACGAGCCAATCGGG CTCTGACACTGTGAT GAGGCGCTGCCTGCC  U  R  G  L  F   G  N  G  V  S   N  E  P  I  G   L  U  H  C  D   E  A  L  P  A  TCCTGACAGTGACAG GGACTGATGGGCGAC TCCACCAATCACATC AGGGAGACCTGACAG TGACGCTCGCCCCCC  S  U  Q  U  Q   G  L  M  G  D   S  T  N  H  I   R  E  T  U  Q   U  R  S  P  P  GCTGACTGACAGCAG CCTCCGCCAGTGATG TCAGCCTGATCCCCG GGTGTTGAAACCTGA GGCTGAGAGCAGCTG  A  D  U  Q  Q   P  P  P  V  M   S  A  U  S  P   G  V  E  T  U   G  U  E  Q  L  TAAGCAGGGCCATGG CTCTGTGAAAGTTAC CTTGCTTATAGGCCT CATATTAACCTCTGT GTGACCAACACCGGG  *    GAAAAGCGCTCATAC CATCTTACCAACTAG TATTGCTCCGGTACT GTAAAGCCACACTTG ATAGAGAGGGATGGA GGAATACATTGGGTC TGGTAGAAGAATGCT AAGCCTAAACCTATT GCGATATGGGGAATA GGATGGTATTTGAGA TGTACCCAAAGGGAT AGTATGACTTCAAGG AAGGTAGCCTCCACC CTCTCGCTCTCTGTC GGTGCGTTGGTGTCA CCCTCCATGGAGTGT ATGGGGTGACTATGT TGATGTTTCTCCTCC TCCGTTTTGCTCCCT CCATCCAAAATGAAG GCAGGCACAGGAACT ACCCTCTAGTGGTGT CTGTCTGATGTCCGG CTGGGGAGAGGGGAG GGGAGGGCCATACAA TATGACCACCCCCTA AAGTTCAAAGTTCAG AATATCGAGAGCTTT GGAAGGAGTATGAAG GAGTAGCGGCCAATG TTGAAATAAACTGAC ATCATGGTCAGAAAT GCAAGATTTGAACCA AAGTTAGACATTGTT ATATTCAGCGTTAGT TAGACATTGTTATAT TCAGCGTTAGTTAGA CATTGTTATATTCAG CGTTAGTTAGACATT GTTATATTCAGCGTT AGTTAGACATTGTTA TATTCAGCCTTAGTT AGACATTGTTATACT CAGCGTTAGTTAGAC ATTGTTATATCAGCG TTAGTTAGACATTGT TATATTCAGCGTTAG TTAGACATTGTTATA TTCAGTGTTAGCAAA ACAAATTTGGTTAAA ATACATTATTGATAG AGTAAGATTTTTCTA TTATGACCCCATCAA CATTTTCAACTTTTT CTTCAACTAAAAATC TAATGTCACTCCTTT CAAGCAACAACATTT ATTTTTACCTTTGAA TTAACGCTGTGTTTA TTAAAGCAGTAGTGT GTTTTGTAGACAAAA TTCTATATTCATAGC GTACAGCTGTAATAA TGTGCGTTTAGTTTT CCATTTTTCTGTCAC TGTTATTGCTGTTGG CAATATTACACACAT ATCCATAATGCACTG TTTGTGAACTAACAA AACAAAGTGACCACT TTTCAACTAACAAAA AAACCAGCTGGAATG AAGGTGGGAAGAGAG AAATATTGAATATAC TGTACTTAATGAAGG TCTCTGGGGAAGACT ACAGTCACCTAGAAC ACTCTATGCTTTGTA CAGAACAAATTCTTT CTGACTTTGGAGTGT AGGTGGTGGAACATG TAAGAA |
| --- | --- |
|  |  |

**S2 Figure: Nucleotide and deduced amino acid sequences of Atlantic salmon SelPa2**. The cDNA sequence was obtained by PCR. The start and stop codons for translation are highlighted in red. The TGA codon for Sec (U) is highlighted in green. The primer binding sites for PCR amplification are boxed. Intron positions are indicated by red arrowheads. A predicted signal peptide is highlighted in green. The predicted SECIS element is highlighted in yellow and boxed. A region of predicted tandem repeats (9 copies of 26 bp repeats with a consensus sequence of AGTTAGACATTGTTATATTCAGCGTT) is shaded in grey.
