# Supplementary material for: Four selenoprotein P genes exist in salmonids: Analysis of their origin and expression following Se supplementation and bacterial infection
Source: PLoS One. 2018 Dec 20;13(12):e0209381. doi: 10.1371/journal.pone.0209381 (PMC6301783; doi:10.1371/journal.pone.0209381)
Supplement: S3 Fig — (DOCX) [file pone.0209381.s003.docx]

S3 Figure S3

| 1  76 151 1 226 10 301 35 376 60 451 85 526 110 601 135 676 160 751 185 826 901 976 1051 1126 1201 | AGAAATGCAACACAC AGCCAGATTACAGAA AGAGGTAGAGGAAAT GAAAAAGCGGATGTT CAGACTGGATAGAAT AGAGAGGGGCCTCCA GACATTTCTGTATGA AGACCAGATCCGTGC CCTGACACTGACCAA GCGTTCCCGGAGAGC TGTGTGGTCCCACGA GACAGTGCTGAAGGC CCGTGAGGTTGGGAG AAGATGATGCAGGGT CTCTTTACTCTGAGG   M  M  Q  G   L  F  T  L  R  CTGTGTGCTGCTCTG CCAGGGCTCCTATGG GCATCGCCTCTGTTA GTAGAAGGGGACAAT GATGCCTCCAAGATC  L  C  A  A  L   P  G  L  L  W   A  S  P  L  L   V  E  G  D  N   D  A  S  K  I  TGTAAGCCGGCACCG CGCTGTGAGATCAAG GGCCATGGGGCCCCC ATGAAGGGGCTGCTT GGAAATTTAGTTGTT  C  K  P  A  P   R  C  E  I  K   G  H  G  A  P   M  K  G  L  L   G  N  L  V  V  CTGGCTCTACTGAAA GCCTGCTGACACTTC TGCCTCACACAGGCC TCCAAACTAGAAGGC CTGCATGACAAGCTG  L  A  L  L  K   A  C  U  H  F   C  L  T  Q  A   S  K  L  E  G   L  H  D  K  L  CTGCGCAGCAACCTG ACAGACATGTCTTTC CTCATTGTGAATGAA CGGGAGGTCCAGTCC TGAGCCATGTACTGG  L  R  S  N  L   T  D  M  S  F   L  I  V  N  E   R  E  V  Q  S   U  A  M  Y  W  GAACTGAAGAGGAGG GCCACCCCGGACATC CCTGTCTACCAACAG GCCCAGCTACAGGAT GATGTCTGGGAGGCC  E  L  K  R  R   A  T  P  D  I   P  V  Y  Q  Q   A  Q  L  Q  D   D  V  W  E  A  CTATATGGAAACAAG GACGACTTCCTGGTA TATGACAGGAATCCA AATGGAGTTACTCAG CTGGGTGGAACAGCA  L  Y  G  N  K   D  D  F  L  V   Y  D  R  N  P   N  G  V  T  Q   L  G  G  T  A  GACAGCGAAACGAGT CTCTGAGCAGCGCAG GAATGGCTGTTAACA AGACAGACACTACAG TGTCAGATTGAAGTC  D  S  E  T  S   L  U  A  A  Q   E  W  L  L  T   R  Q  T  L  Q   C  Q  I  E  V  GCTACTGTCAGCAAC CCAGTTCCAGATGTT GTCTGAGGGGGGCGG TAACATGCCAAACAT ACATCACCAGCAGCA  A  T  V  S  N   P  V  P  D  V   V  U  G  G  R   *    TCATCAGCACCACAA CCCTGGGTCAGACAC AGATAAACAGGACTC CGATTAACATAATCT GTGTCGTTGCGGAGA CGAGTAACAGTTAGT TAGCATGGTGGTGGG GGCTTGTGTCAGTGA CTTGACTGTCTGATA AATAGTTTTGTATGA ATTCAGTTTGATTTT GTCTGTGCCTGTCAA TTGTATCTGATAAAG TAAACTTGTACAGTA AACTGGAGTAATTTG GCCCTTGGAATACAG TATGTCTTCCCTACA TGTGACATGTCGGGT GACATGTTCTACTCT CTGCTATGAAGTCTG CAGGGCAAACCTTAC TGAGGTGTCTGTAGA CAGATGCTGTGCTGA GGAAAACGGGACTGA CAGTCACTGAACCCA TGCAGGGCAGGGTGC TGGATGTCAGGGTAG ACTTAGGCATCCAAA ATGTA |
| --- | --- |

**S3 Figure:** **Nucleotide and deduced amino acid sequences of rainbow trout SelPb2.** The cDNA sequence was obtained by PCR. The start and stop codons for the main open reading frame (ORF) and the immediate upstream stop codon of the main ORF are highlighted in red. The TGA codon for Sec (U) is highlighted in green. The primer binding sites for PCR amplification are boxed. Intron positions are indicated by red arrowheads. A predicted signal peptide is highlighted in green. The predicted SECIS element is highlighted in yellow and boxed.

| 1  76 151 1 226 10 301 35 376 60 451 85 526 110 601 135 676 160 751 185 826 901 976 1051 1126 1201 | AGAAATGCAACACAC AGCCAGATTACAGAA AGAGGTAGAGGAAAT GAAAAAGCGGATGTT CAGACTGGATAGAAT AGAGAGGGGCCTCCA GACATTTCTGTATGA AGACCAGATCCGTGC CCTGACACTGACCAA GCGTTCCCGGAGAGC TGTGTGGTCCCACGA GACAGTGCTGAAGGC CCGTGAGGTTGGGAG AAGATGATGCAGGGT CTCTTTACTCTGAGG   M  M  Q  G   L  F  T  L  R  CTGTGTGCTGCTCTG CCAGGGCTCCTATGG GCATCGCCTCTGTTA GTAGAAGGGGACAAT GATGCCTCCAAGATC  L  C  A  A  L   P  G  L  L  W   A  S  P  L  L   V  E  G  D  N   D  A  S  K  I  TGTAAGCCGGCACCG CGCTGTGAGATCAAG GGCCATGGGGCCCCC ATGAAGGGGCTGCTT GGAAATTTAGTTGTT  C  K  P  A  P   R  C  E  I  K   G  H  G  A  P   M  K  G  L  L   G  N  L  V  V  CTGGCTCTACTGAAA GCCTGCTGACACTTC TGCCTCACACAGGCC TCCAAACTAGAAGGC CTGCATGACAAGCTG  L  A  L  L  K   A  C  U  H  F   C  L  T  Q  A   S  K  L  E  G   L  H  D  K  L  CTGCGCAGCAACCTG ACAGACATGTCTTTC CTCATTGTGAATGAA CGGGAGGTCCAGTCC TGAGCCATGTACTGG  L  R  S  N  L   T  D  M  S  F   L  I  V  N  E   R  E  V  Q  S   U  A  M  Y  W  GAACTGAAGAGGAGG GCCACCCCGGACATC CCTGTCTACCAACAG GCCCAGCTACAGGAT GATGTCTGGGAGGCC  E  L  K  R  R   A  T  P  D  I   P  V  Y  Q  Q   A  Q  L  Q  D   D  V  W  E  A  CTATATGGAAACAAG GACGACTTCCTGGTA TATGACAGGAATCCA AATGGAGTTACTCAG CTGGGTGGAACAGCA  L  Y  G  N  K   D  D  F  L  V   Y  D  R  N  P   N  G  V  T  Q   L  G  G  T  A  GACAGCGAAACGAGT CTCTGAGCAGCGCAG GAATGGCTGTTAACA AGACAGACACTACAG TGTCAGATTGAAGTC  D  S  E  T  S   L  U  A  A  Q   E  W  L  L  T   R  Q  T  L  Q   C  Q  I  E  V  GCTACTGTCAGCAAC CCAGTTCCAGATGTT GTCTGAGGGGGGCGG TAACATGCCAAACAT ACATCACCAGCAGCA  A  T  V  S  N   P  V  P  D  V   V  U  G  G  R   *    TCATCAGCACCACAA CCCTGGGTCAGACAC AGATAAACAGGACTC CGATTAACATAATCT GTGTCGTTGCGGAGA CGAGTAACAGTTAGT TAGCATGGTGGTGGG GGCTTGTGTCAGTGA CTTGACTGTCTGATA AATAGTTTTGTATGA ATTCAGTTTGATTTT GTCTGTGCCTGTCAA TTGTATCTGATAAAG TAAACTTGTACAGTA AACTGGAGTAATTTG GCCCTTGGAATACAG TATGTCTTCCCTACA TGTGACATGTCGGGT GACATGTTCTACTCT CTGCTATGAAGTCTG CAGGGCAAACCTTAC TGAGGTGTCTGTAGA CAGATGCTGTGCTGA GGAAAACGGGACTGA CAGTCACTGAACCCA TGCAGGGCAGGGTGC TGGATGTCAGGGTAG ACTTAGGCATCCAAA ATGTA |
| --- | --- |

**Figure S3:** **Nucleotide and deduced amino acid sequences of rainbow trout SelPb2.** The cDNA sequence was obtained by PCR. The start and stop codons for the main open reading frame (ORF) and the immediate upstream stop codon of the main ORF are highlighted in red. The TGA codon for Sec (U) is highlighted in green. The primer binding sites for PCR amplification are boxed. Intron positions are indicated by red arrowheads. A predicted signal peptide is highlighted in green. The predicted SECIS element is highlighted in yellow and boxed.
