# Supplementary material for: Four selenoprotein P genes exist in salmonids: Analysis of their origin and expression following Se supplementation and bacterial infection
Source: PLoS One. 2018 Dec 20;13(12):e0209381. doi: 10.1371/journal.pone.0209381 (PMC6301783; doi:10.1371/journal.pone.0209381)
Supplement: S4 Fig — (DOCX) [file pone.0209381.s004.docx]

S4 Figure

| 1 77 152 1 227 4 302 29 377 54 452 79 527 104 602 129 677 752 827 902 977 1052 1127 1202 1277 1352 1427 1502 1577 | ACAGAACACTGCTGGAACAAGAAATGCAACA CACAGCCAGATTACA GAAAGAGGTAGAGGA AATGAAAAAGCGGAT GTTCAGACTGGACAG AATAGAGAGGGGCCT TCAGACATTTCTGTA TGAAGACCAGATCCG CGCCCTGACACTGAC CAAGCGTTCCCGGAG AGCCGTGTGGTCCCA CGAGACAGTGCTGAA GGCCCGTGAGGTTGG GAGAAGATGATGCAG  M  M  Q   GGTCTCTTTACTCTG AGACTGTGTGCTGCT CTGCCAGGGCTCCTA TGGGCATCGCCTCTG TTAGTAGAAGGGGAC  G  L  F  T  L   R  L  C  A  A   L  P  G  L  L   W  A  S  P  L   L  V  E  G  D  ACTGATGCCTCCAAG ATCTGCAAGCCGGCA CCGCGCTGGGAGATC AAGGGCCATGGGGCC CCCATGAAGGGGCTG  T  D  A  S  K   I  C  K  P  A   P  R  W  E  I   K  G  H  G  A   P  M  K  G  L  CTGGGAAATGTAGTC GTTCTGGCTCTACTG AAAGCCAGCTGACAC TTCTGCCTCACACAG GCCTCAAAGAGGTCC  L  G  N  V  V   V  L  A  L  L   K  A  S  U  H   F  C  L  T  Q   A  S  K  R  S  AGTCCTGAGCCATGT ATTGTGAACTGAAGA GGAGCCCCCCCCCCC CCCCCGGGCATCCCT GTCTACCAACAGGCC  S  P  E  P  C   I  V  N  U  R   G  A  P  P  P   P  P  G  I  P   V  Y  Q  Q  A  CAGCTATATGGAAAC AAGGACGACTTCCTG GTATATGACAGAGAT CTTTGTAATTGGTGT GACACCTCATTCATG  Q  L  Y  G  N   K  D  D  F  L   V  Y  D  R  D   L  C  N  W  C   D  T  S  F  M  TGGCCGTGGATCTCT CTCCTCTCGCTGATA GATGTGGGAGACTGA CGTGCCACATAGTCC TGCCCTACAGCTTCC  W  P  W  I  S   L  L  S  L  I   D  V  G  D  U   R  A  T  *    TCCACTACCCCAATA TAGATGCAGCTGTCA GAGCCACTTACCACA AGGACATCTATGGCA ACTGCACCATGAGTG  AAAGCACACTTCTAA AGGGAATTACAGACA TTTTCATTTCTGTCT GGCTCGTCATTAGAA AACACTGTCTGTCTG CTGTAGTGGCCCCAT TGGAATAGAATGTCT AGAACAGGAACACCC CTACCTATAATGGGA ACTGATTCTGAATAA  TAATGAGATCGCTCT GCTGCTCTCCCAGGT GATGTCTTTTTAGCT CGTACCTATTTTGTT AACTTTAGTGTGTTC TACTGTTACGCAGGA ATCCAAATGGAGTTA CTCAGCTGGGTGGAA CAGCAGACAGCGGAA CGAGTCTCTGAGCAG CGTAGGAATGGCTGT TAACGAGACAAACAC TACAGTGAGGCAGAT TGAAGTCGCTACTGT CAGCAACCCAGTTCC AGATGTTGTCTGAGG GGGGTGGTAACATGC CAAACATACATCACC AGCAGCATCGTCAGC ACCACCACCACCATG GGTCAGACACCGATA AACAGGACTCCTATT GACATCATCTGTGTA GTTGCTGAGACGAGT AATAGTTAGCATGGT GGTGGGGGCTTGTGT CAGTGACTTGACTGT CTGATAAATAGTTTT GTGTGAATTCAGTTC GATTTTGTCTGTGCC TGTCAATTGTATCTG ATAAAGCAAACCTGT ACAGTAAACTGGAGT CATTTGGCCCTTGGA ATACAGTCCCTACAT GTGGTCGGGTGACAT GTTCTACTCTCTGCT ATGAAGTCTGCAGGG CAAACCTTACTGAGG TGTCTGTAGACTGAT GCTGTGCTGAGGAAA ACGGGACTGACAGTC ACTGAACCCATGCAG GGCAGTGTGCTGGAC TTCAGGGTATACTTA GGCATCCAAAATGTA GGTTCCAGGTTGTTC CATGGTCAGATACG |
| --- | --- |

**S4 Figure:** **Nucleotide and deduced amino acid sequences of Atlantic salmon SelPb2.** The cDNA sequence was obtained by PCR. The start and stop codons for the main open reading frame (ORF) and the immediate upstream stop codon of the main ORF are highlighted in red. The TGA codon for Sec (U) is highlighted in green. The primer binding sites for PCR amplification are boxed. Intron positions are indicated by red arrowheads. A predicted signal peptide is highlighted in green. The predicted SECIS element is highlighted in yellow and boxed.
