# Supplementary material for: Four selenoprotein P genes exist in salmonids: Analysis of their origin and expression following Se supplementation and bacterial infection
Source: PLoS One. 2018 Dec 20;13(12):e0209381. doi: 10.1371/journal.pone.0209381 (PMC6301783; doi:10.1371/journal.pone.0209381)
Supplement: S5 Fig — (DOCX) [file pone.0209381.s005.docx]

S5 Figure

SelPa2_iso1 1 CACACTCAGCAGATCGAGCCTGCTGCAAGGCACCGTGATGAAGGCGGGGCTCAGCCTGCTCCTGGCTCTCTGCCTGCTCCCTGGGGGCGG
SelPa2_iso2 1 CACACTCAGCAGATCGAGCCTGCTGCAAGGCACCGTGATGAAGGCGGGGCTCAGCCTGCTCCTGGCTCTCTGCCTGCTCCCTGGGGGCGG

SelPa2_iso1 91 AGCAGAGAGTGAGGGGGAGGGGACCCGCTGTAAGCCACCACCTGGTTGGAGCATTGGGGAGGTGGAGCCAATGAAGGGGGTTATGGGCCA
SelPa2_iso2 91 AGCAGAGAGTGAGGGGGAGGGGACCCGCTGTAAGCCACCACCTGGTTGGAGCATTGGGGAGGTGGAGCCAATGAAGGGGGTTATGGGCCA

SelPa2_iso1 181 GGTCACGGTGGTGGCCCTCCTCCAGGCCAGCTGATCGTTCTGCTTGGTGCAGGCATCCTTATTGGATGAGCTGCGCCTGAAGCTGGAGGG
SelPa2_iso2 181 GGTCACGGTGGTGGCCCTCCTCCAGGCCAGCTGATCGTTCTGCTTGGTGCAGGCATCCTTATTGGATGAGCTGCGCCTGAAGCTGGAGGG

SelPa2_iso1 271 CCAGGGTCTGGACAATGTGACCTATATGGTGGTGAACCACCAGGGGGAGCAGGCCCAGCACCTTCACACCTTGCTGAGCCAGAAACTGTC
SelPa2_iso2 271 CCAGGGTCTGGACAATGTGACCTATATGGTGGTGAACCACCAGGGGGAGCAGGCCCAGCACCTTCACACCTTGCTGAGCCAGAAACTGTC

SelPa2_iso1 361 TGAGAACATCATATTGTACAAACAGGTACCCAAACAGGATGACGTGTGGCAGGCCCTGGCTGGAAAGAAGGATGACTTCCTCATCTATGA
SelPa2_iso2 361 TGAGAACATCATATTGTACAAACAGGTACCCAAACAGGATGACGTGTGGCAGGCCCTGGCTGGAAAGAAGGATGACTTCCTCATCTATGA

SelPa2_iso1 451 CAGGTGTGGTCGTCTGACCCACCATATCTTCCTCCCCTTTTCCATCCTGGGTACTCCCTACGTAGAGAACGCCATTAAGGAGACCTACTG
SelPa2_iso2 451 CAGGTGTGGTCGTCTGACCCACCATATCTTCCTCCCCTTTTCCATCCTGGGTACTCCCTACGTAGAGAACGCCATTAAGGAGACCTACTG

SelPa2_iso1 541 CCAACGCATCTGTGGGGACTGCACGTATGAGAACACAGAGATCCCAGCAGAGTGCAACAGGATGGTAGAGGTAAAGCCTGAGGGAGAAGA
SelPa2_iso2 541 CCAACGCATCTGTGGGGACTGCACGTATGAGAACACAGAGATCCCAGCAGAGTGCAACAGGATGGTAGAGGTAAAGCCTGAGGGAGAAGA

SelPa2_iso1 631 GAAGCCAGTCACTGGAGGGGATACACCTCACGGTGGACGCGGCCATCATCACCATGGCAATGGGCACGGTCACCATGGCAAAAGCCATGG
SelPa2_iso2 631 GAAGCCAGTCACTGGAGGGGATACACCTCACGGTGGACGCGGCCATCATCACCATGGCAATGGGCACGGTCACCATGGCAAAAGCCATGG

SelPa2_iso1 721 TCACGGTCACCATGGGGAGAGTGATGTGGGGCGCGAACACGGTCGTGGCCATGGGGTGGAGCAGCAGCAGCACCAAAATGGCGCTGAGAG
SelPa2_iso2 721 TCACGGTCACCATGGGGAGAGTGATGTGGGGCGCGAACACGGTCGTGGCCATGGGGTGGAGCAGCAGCAGCACCAAAATGGCGCTGAGAG

SelPa2_iso1 811 GCTCCACCATGGCCAGGCCCATGGCCAAGTGCACGTTGGTCAGGAGCATATGGGTCAGCAGCCCAAGGAGGTGCAGGAAGGGCATATTAT
SelPa2_iso2 811 GCTCCACCATGGCCAGGCCCATGGCCAAGTGCACGTTGGTCAGGAGCATATGGGTCAGCAGCCCAAGGAGGTGCAGGAAGGGCATATTAT

SelPa2_iso1 901 GCAGAGGCCCTGAGTGAAGGGGAGGGCCAGGTGAAAGGCAGAGCTCAGCTGACATTTGAAGGAGGGGTCTGACATAAGTCCCTCCTCCAA
SelPa2_iso2 901 GCAGAGGCCCTGAGTGAAGGGGAGGGCCAGGTGAAAGGCAGAGCTCAGCTGACATTTGAAGGAGGGGTCTGACATAAGTCCCTCCTCCAA

SelPa2_iso1 991 GGTCAGCTGATGCTGACACTGACGGGGGCTGTTTGGCAATGGGGTGCGCAACGAGCCAATCGGGCTCTGACACTGTGATGAGGCGCTGCC
SelPa2_iso2 991 GGTCAGCTGATGCTGACACTGACGGGGGCTGTTTGGCAATGGGGTGCGCAACGAGCCAATCGGGCTCTGACACTGTGATGAGGCGCTGCC

SelPa2_iso1 1081 CGCCTCCTGACAGTGACAGGGACTGATGGGCGACTCCAACAATCACATCAGGGAGACCTGACAGTGACGCTTGCCCCCCACAGACTGACA
SelPa2_iso2 1081 CGCCTCCTGACAGTGACAGGGACTGATGGGCGACTCCAACAATCACATCAGGGAGACCTGACAGTGACGCTTGCCCCCCACAGACTGACA

SelPa2_iso1 1171 GCAGCCTCTGCCAGTGATGTCAGCCTGATCCCCGGGTGTTGAAACCTGAAGCTGAGAGCAGCTGTAAGCAGGGCCATGGCTCTGTGAAAG
SelPa2_iso2 1171 GCAGCCTCTGCCAGTGATGTCAGCCTGATCCCCGGGTGTTGAAACCTGAAGCTGAGAGCAGCTGTAAGCAGGGCCATGGCTCTGTGAAAG

SelPa2_iso1 1261 TTATCTTGCTTATAGGCCTCATATTAACATCTGTGAGACCAACACCGGGGCAAAGCGCTCATACCATCTTACCAACTAGTATTGCTCCGG
SelPa2_iso2 1261 TTATCTTGCTTATAGGCCTCATATTAACATCTGTGAGACCAACACCGGGGCAAAGCGCTCATACCATCTTACCAACTAGTATTGCTCCGG

SelPa2_iso1 1351 TACTGTAAAGCCACACTTGATAGAGAGGGATGGAGGAATACATTGGGTCTGGTAGAAGAATGCTAAGCCTAAACCTGTTACGATATGGGG
SelPa2_iso2 1351 TACTGTAAAGCCACACTTGATAGAGAGGGATGGAGGAATACATTGGGTCTGGTAGAAGAATGCTAAGCCTAAACCTGTTACGATATGGGG

SelPa2_iso1 1441 AATAGGATAGTATTTGAGATGTACCCAAAGGGATAGTATGACATCAAGGAAGGTAGCCTCCACCCTCTCTTTCTCTGTCGGTGCATTGGT
SelPa2_iso2 1441 AATAGGATAGTATTTGAGATGTACCCAAAGGGATAGTATGACATCAAGGAAGGTAGCCTCCACCCTCTCTTTCTCTGTCGGTGCATTGGT

SelPa2_iso1 1531 GTCACCATCCCGGGAGTGTATTGGGTGACTATTTTGATGTTTCTCCCCCTCCGTTTTGCTCCCTCCATCCAAAATGAAGGTAGGCACAGA
SelPa2_iso2 1531 GTCACCATCCCGGGAGTGTATTGGGTGACTATTTTGATGTTTCTCCCCCTCCGTTTTGCTCCCTCCATCCAAAATGAAGGTAGGCACAGA

SelPa2_iso1 1621 AACTACGCTGTAGTGGTGTCTGTCTGATGTCCGGCTGGGGAGAGGGGAGGGTGAGGGCCATACAATATGACCACCCCCTTAATTCAAAGT
SelPa2_iso2 1621 AACTACGCTGTAGTGGTGTCTGTCTGATGTCCGGCTGGGGAGAGGGGAGGGTGAGGGCCATACAATATGACCACCCCCTTAATTCAAAGT

SelPa2_iso1 1711 TCACAATATCGAGAGCTTTAGAAGGAGTATGAAGGAGTAGCGTCCAATGTTGAAATAAACTGAAATCATGGCCAGAAATGCAAGATTTGA
SelPa2_iso2 1711 TCACAATATCGAGAGCTTTAGAAGGAGTATGAAGGAGTAGCGTCCAATGTTGAAATAAACTGAAATCATGGCCAGAAATGCAAGATTTGA

SelPa2_iso1 1801 ACCAAAGTTAGACATTGTTATATTCAGCGTTAGTTAGACATTGTTATACTCAGCGTTAGTTAGACATTGTTACATTCAGCGTTAGTTAGA
SelPa2_iso2 1801 ACCAAAGTTAGACATTGTTATATTCAGCGTTAGTTAGACATTGTTATACTCAGCGTTAGTTAGACATTGTTACATTCAGCGTTAGTTAGA

SelPa2_iso1 1891 CATTGTTATACTCAGCGTTAGTTAGACATTGTTATACTCAGCGTTAGTTAGACATTGTTATACTCAGCGTTAGTTAGACATTGTTATACT
SelPa2_iso2 1891 CATTGTTATACTCAGCGTTAGTTAGACATTGTTATACTCAGCGTTAGTTAGACATTGTTATACTCAGCGTTAGTTAGACATTGTTATACT

SelPa2_iso1 1981 CAGCGTTAGTTAGACATTGTTATATTCAGCGTTAGTTAGACATTGTTATACTCAGCGTTAGTTAGACATTGTTATACTCAGCGTT-----
SelPa2_iso2 1981 CAGCGTTAGTTAGACATTGTTATATTCAGCGTTAGTTAGACATTGTTATACTCAGCGTTAGTTAGACATTGTTATACTCAGCGTTAGTTA

SelPa2_iso1 2064 -------------------------------------------------------------------------AGTTAGACATTGTTATA
SelPa2_iso2 2071 GACATTGTTATATTCAGCGTTAGTTAGACATTGTTATACTCAGCGTTAGTTAGACATTGTTATATTCAGCGTTAGTTAGACATTGTTATA

SelPa2_iso1 2083 TTCAGCGTTAGTTAGACATTGTTATACTCAGCGTTAGTTAGACATTGTTATATTCAGCGTTAGTTAGACATTGTTATACTCAGCGTTAGT
SelPa2_iso2 2161 TTCAGCGTTAGTTAGACATTGTTATACTCAGCGTTAGTTAGACATTGTTATATTCAGCGTTAGTTAGACATTGTTATACTCAGCGTTAGT

Figure 5 Continued,

SelPa2_iso1 2173 TAGACATTGTTATACTCAGCGTTAGTTAGACATTGTTATATTCAGCGTTAGTTAGACATTGTTATATTCAGCGTTAGTTAAACATTGTTA
SelPa2_iso2 2251 TAGACATTGTTATACTCAGCGTTAGTTAGACATTGTTATATTCAGCGTTAGTTAGACATTGTTATATTCAGCGTTAGTTAAACATTGTTA

SelPa2_iso1 2263 TATTCAGTGTTAGTTAGATATTGTTATATTCAGCGTTAGTTAAACATTGTTATATTCAGTGTTAACAAAACACATTTGAATAAAATACAT
SelPa2_iso2 2341 TATTCAATGTTAGTTAGATATTGTTATATTCAGCGTTAGTTAAACATTGTTATATTCAGTGTTAACAAAACACATTTGAATAAAATACAT

SelPa2_iso1 2353 TATTGATAGAGTAAGATTTTTCTATTATGACCCCATCAACCTTCTCAACTTTTTCTTCAACTAAAAATCTAATGTCACTCCTTTCAAGCA
SelPa2_iso2 2431 TATTGATAGAGTAAGATTTTTCTATTATGACCCCATCAACCTTTTCAACTTTTTCTTCAACTAAAAATCTAATGTCACTCCTTTCAAGCA

SelPa2_iso1 2443 ACAACATTTATTTGTACCTTTGAATTAACGCTGTGTTTATTAAAGCAGTAGTGTGTTTTGTAGACAAAATTCAATATTCATAGTGTACAG
SelPa2_iso2 2521 ACAACATTTATTTGTACCTTTGAATTAACGCTGTGTTTATTAAAGCAGTAGTGTGTTTTGTAGACAAAATTCAATATTCATAGCGTACAG

SelPa2_iso1 2533 CAGTGATAATGTGTGTTTAGTTTTCCATGTTTCTGTCACTGTTATTGCTGTTGGCAATATTACACATATCTATAATGCACTGTTCATGAA
SelPa2_iso2 2611 CTGTGATAATGTGTGTTTAGTTTTCCATGTTTCTGTCACTGTTATTGCTGTTGGCAATATT-----------------------------

SelPa2_iso1 2623 CTAACAAAACAAAGTGACCACTTTTCAACTAACAAAATATATTACACACATATCTATAATGCACTGTTCATGAACTAACAAAACAAAGTG
SelPa2_iso2 2672 ---------------------------------------------ACACATATCTATAATGCACTGTTCATGAACTAACAAAACAAAGTG

SelPa2_iso1 2713 ACCGCTTTTCAACCAACAAAAAAACAGCTGGAATGAAGGTGGGTAGAGAGAAATATTGAATATACCGTACTTAATGAAGGTCTCTGGGGA
SelPa2_iso2 2717 ACCGCTTTTCAACCAACAAAAAAACAGCTGGAATGAAGGTGGGTAGAGAGAAATATTGAATATACTGTACTTAATGAAGGTCTCTGGGGA

SelPa2_iso1 2803 AGACTACGGTCACCTAGAACACCC

SelPa2_iso2 2807 AGACTACGGTCACCTAGAACACCC

**S5 Figure: Alignment of the two trout SelPa2 cDNA sequences cloned in this study. The primer binding sites** for PCR cloning are in green. The start and stop codons of the main open reading frame (ORF) are in red. The main difference between the two cDNA sequences lies in the 3’-UTR at two repeat regions highlighted in red and blue background, respectively. The first repeat region has 29 copies of a 26 bp consensus sequence (AGTTAGACATTGTTATATTCAGCGTT) for isoform 1, but 29 copies for isoform 2.
