# Supplementary material for: Four selenoprotein P genes exist in salmonids: Analysis of their origin and expression following Se supplementation and bacterial infection
Source: PLoS One. 2018 Dec 20;13(12):e0209381. doi: 10.1371/journal.pone.0209381 (PMC6301783; doi:10.1371/journal.pone.0209381)
Supplement: S6 Fig — cDNA sequence alignment of salmonid SelPb paralogues (A) and schematic gene organisation of the coding region (B). (DOCX) [file pone.0209381.s006.docx]

S6 Figure

**A.**

Trout_SelPb1 18 ----------------------------GGAGGAC**ATG**ATGCAGGGTCTCTTGACTCTGTGGCTGTGTGCTGCTCTGCCAGGGCTCCTATGCGCATCGCC
Salmon_SelPb1 119 ----------------------------GGAGGAC**ATG**ATGCAGGGTCTCTTGACTCTGTGGCTGTGTGCTGCTCTGCCAGGGCTCCTATGCGCATCGCC
Trout_SelPb2 164 GAGACAGTGCTGAAGGCCCGTGAGGTTGGGAGAAG**ATG**ATGCAGGGTCTCTTTACTCTGAGGCTGTGTGCTGCTCTGCCAGGGCTCCTATGGGCATCGCC
Salmon_SelPb2 184 GAGACAGTGCTGAAGGCCCGTGAGGTTGGGAGAAG**ATG**ATGCAGGGTCTCTTTACTCTGAGACTGTGTGCTGCTCTGCCAGGGCTCCTATGGGCATCGCC

Trout_SelPb1 90 TCTGTTAGTAGAAGGGGACAATGATGCCTCCAAGATCTGCAAGCCTGCACCGCACTGGGAGATCAAGGGCCATGGGGCCCCCATGAAGGAGCTGCTGGGA
Salmon_SelPb1 191 TCTGTTAGTAGAAGGGGACAATGATGCCTCCAAGATCTGCAAGACTGCACCGCACTGGGAGATCAAGGGCCATGGGGCCCCCATGAAGGAGCTGCTGGGA
Trout_SelPb2 264 TCTGTTAGTAGAAGGGGACAATGATGCCTCCAAGATCTGTAAGCCGGCACCGCGCTGTGAGATCAAGGGCCATGGGGCCCCCATGAAGGGGCTGCTTGGA
Salmon_SelPb2 284 TCTGTTAGTAGAAGGGGACACTGATGCCTCCAAGATCTGCAAGCCGGCACCGCGCTGGGAGATCAAGGGCCATGGGGCCCCCATGAAGGGGCTGCTGGGA

Trout_SelPb1 190 AATGTAGTTGTTCTGGCTCTGCTGAAAGCAAGCTGACACTTCTGCCGCACACAGGCCTCCAAACTAGGAGGCCTGCGTGACAAGCTGCTCCGCAGCAACC
Salmon_SelPb1 291 AATGTAGTTGTTCTGGCTCTGCTGAAAGCAAGCTGACACTTCTGCCGCACACAGGCCTCCAAACTAGGAGGCCTGCGTGACAAGCTGCTCCACAGCAACC
Trout_SelPb2 364 AATTTAGTTGTTCTGGCTCTACTGAAAGCCTGCTGACACTTCTGCCTCACACAGGCCTCCAAACTAGAAGGCCTGCATGACAAGCTGCTGCGCAGCAACC
Salmon_SelPb2 384 AATGTAGTCGTTCTGGCTCTACTGAAAGCCAGCTGACACTTCTGCCTCACACAGGCCTCCAA--------------------------------------

Trout_SelPb1 290 TGACAGACGTGTCGTTCCTCATCGTGAACGAGAGGGAAGCCCAGTCCAGAGCCATGTACTGGGAGCTGAAGAGGAGGGCACC------CCCGGGCATCCC
Salmon_SelPb1 391 TGACAGACGTGTCGTTCCTCATCGTGAACGAGAGGGAGGCCCAGTCCAGAGCCATGTACTGGGAGCTGAAGAGGAGGGCCCC------CCCGGGCATCCC
Trout_SelPb2 464 TGACAGACATGTCTTTCCTCATTGTGAATGAACGGGAGGTCCAGTCCTGAGCCATGTACTGGGAACTGAAGAGGAGGGCCAC------CCCGGACATCCC
Salmon_SelPb2 446 -----------------------------------GAGGTCCAGTCCTGAGCCATGTATTGTGAACTGAAGAGGAGCCCCCCCCCCCCCCCGGGCATCCC

Trout_SelPb1 384 TGTCTACCAACAGGCCCCGCTACAGGACGACGTCTGGGAGGCCTTGGATGGAGACAAGGACGACTTCCTGGTATATGACAG-------------------
Salmon_SelPb1 485 TGTCTACCAACAGGCCCCGCTACAGGACGACGTCTGGGAGGCCTTGGACGGAGACAAGGACGACTTCCTGGTATATGACAG-------------------
Trout_SelPb2 558 TGTCTACCAACAGGCCCAGCTACAGGATGATGTCTGGGAGGCCCTATATGGAAACAAGGACGACTTCCTGGTATATGACAG-------------------
Salmon_SelPb2 511 TGTCTACCAACAGGCCCAG------------------------CTATATGGAAACAAGGACGACTTCCTGGTATATGACAGAGATCTTTGTAATTGGTGT

Trout_SelPb1 465 ----------------------------------------------ATGTGGGAGACTGACGTTCCACATAGTCCTACCCTACAGCTTCCTCCACTACCC
Salmon_SelPb1 566 ----------------------------------------------ATGTGGGCGACTGACGTTCCACATAGTCCTACCCTACAGCTTCCTCCACTACCC
Trout_SelPb2 639 ----------------------------------------------------------------------------------------------------
Salmon_SelPb2 587 GACACCTCATTCATGTGGCCGTGGATCTCTCTCCTCTCGCTGATAGATGTGGGAGACTGACGTGCCACA**TAG**TCCTGCCCTACAGCTTCCTCCACTACCC

Trout_SelPb1 519 CTACATAGAGGCAGCCGTCAGAGCCACCTACCACAAGGACATCTGTGGCAACTGCACC------------------------------------------
Salmon_SelPb1 620 CTACATAGAGGCAGCCGTCAGAGCCACCTACCACAAGGACATCTGTGGCAACTGCACC------------------------------------------
Trout_SelPb2 639 ----------------------------------------------------------------------------------------------------
Salmon_SelPb2 687 CAATATAGATGCAGCTGTCAGAGCCACTTACCACAAGGACATCTATGGCAACTGCACCATGAGTGAAAGCACACTTCTAAAGGGAATTACAGACATTTTC

Trout_SelPb1 577 ----------------------------------------------------------------------------------------------------
Salmon_SelPb1 678 ----------------------------------------------------------------------------------------------------
Trout_SelPb2 639 ----------------------------------------------------------------------------------------------------
Salmon_SelPb2 787 ATTTCTGTCTGGCTCGTCATTAGAAAACACTGTCTGTCTGCTGTAGTGGCCCCATTGGAATAGAATGTCTAGAACAGGAACACCCCTACCTATAATGGGA

Trout_SelPb1 577 ----------------------------------------------------------------------------------------------------
Salmon_SelPb1 678 ----------------------------------------------------------------------------------------------------
Trout_SelPb2 639 ----------------------------------------------------------------------------------------------------
Salmon_SelPb2 887 ACTGATTCTGAATAATAATGAGATCGCTCTGCTGCTCTCCCAGGTGATGTCTTTTTAGCTCGTACCTATTTTGTTAACTTTAGTGTGTTCTACTGTTACG

Trout_SelPb1 577 GTGGACTCCAACACAACCTCCTCAGCTGGGTGGAACAGCACCCGGAGAAACGAGACACTGAGCAGTTCAGAGATGCGTGTTAATGAGACAGACAG-----
Salmon_SelPb1 678 GTGGACTCCAACACAACCTCCTCAGCTGGGTGGAACAGCTCCCAGAGAAATGAGACACTGAGCAGTTCAGAGATGCGTGTTAACGAGACAGACAGCACAG
Trout_SelPb2 639 ---GAATCCAAATGGAGTTACTCAGCTGGGTGGAACAGCAGACAGCGAAACGAGTCTCTGAGCAGCGCAGGAATGGCTGTTAACAAGACAGACAC-----
Salmon_SelPb2 987 CAGGAATCCAAATGGAGTTACTCAGCTGGGTGGAACAGCAGACAGCGGAACGAGTCTCTGAGCAGCGTAGGAATGGCTGTTAACGAGACAAACAC-----

Trout_SelPb1 672 -------TACAGTGAGGTCTATTGATGTCGATACCGTCAGCAACCCAGTTCCAAGTGATGGACCCCAGATGTCAGCTGAGGGGGGTGGTAACATGTCACA
Salmon_SelPb1 778 TGGTTGATACAGTGAGGTCTACTGATGTCGATACCGTCAGCAACCCAGTTGCAAGTGATGGACGCCAGATGTCGTCTGAGGGGGGTGGTAACATGTCACA
Trout_SelPb2 731 -------TACAGTG--TCAGATTGAAGTCGCTACTGTCAGCAACCCAGTT--------------CCAGATGTTGTCTGAGGGGGGCGG**TAA**CATGCCAAA
Salmon_SelPb2 1082 -------TACAGTGAGGCAGATTGAAGTCGCTACTGTCAGCAACCCAGTT--------------CCAGATGTTGTCTGAGGGGGGTGGTAACATGCCAAA

Trout_SelPb1 765 CATACATCATCAGCATCATCAGTACCCCCACCACCACCAGCAGCAG---------------------CATCAGCACCACCACAATCATGGGTCAGATGCA
Salmon_SelPb1 878 CATACCTCACCAGCATCATCAGTACCCCCACCACCAGCATCATCAGTACCCCCACCATCATCACTATCATCAGCACCACTGAAATCATGGGTCAGACGCA
Trout_SelPb2 808 CATACATCACCAGCAGCAT------------------------------------------------CATCAGCAC---CACAACCCTGGGTCAGACACA
Salmon_SelPb2 1161 CATACATCACCAGCAGCAT------------------------------------------------CGTCAGCACCACCACCACCATGGGTCAGACACC

Trout_SelPb1 844 GATAAACAGGACTCCAAT**TAA**CATTGTCTGTGTCGTTTCTGAGATGAGTAATTGTTAATTAGCATGGTGGTGGTGGTGGCTTGTGTCAGTGACTTGACAG
Salmon_SelPb1 978 GATAAACAGGACTCCAAT**TAA**CATTGTCTGTGTCGTTTCTGAGATGAGTAATTGTTATTTAGCATGGTGGTGGTGGTGGCTTGTGTCAGTGACTTGACCG
Trout_SelPb2 857 GATAAACAGGACTCCGATTAACATAATCTGTGTCGTTGCGGAGACGAGTAACAGTTAGTTAGCA---TGGTGGTGGGGGCTTGTGTCAGTGACTTGACTG
Salmon_SelPb2 1213 GATAAACAGGACTCCTATTGACATCATCTGTGTAGTTGCTGAGACGAGTAA----TAGTTAGCA---TGGTGGTGGGGGCTTGTGTCAGTGACTTGACTG

Trout_SelPb1 944 TCCGATAAATAGTTTTGTATGAATTAAATTAGATTTTGTCTGTGCCTGTCAATTGTGTCTGATAAGGCAAATCTGTACAGTAAACTGGAATTATTTAGGC
Salmon_SelPb1 1078 TCCGATGAATAGTTTTGTATGAATTCAATTTGATTTTGTCTGTATCTGTCAAATGTGTCTGATAAGGCAAACCTGTACAGTAAACTGGAATTATTTAGGC
Trout_SelPb2 954 TCTGATAAATAGTTTTGTATGAATTCAGTTTGATTTTGTCTGTGCCTGTCAATTGTATCTGATAAAGTAAACTTGTACAGTAAACTGGAGTAATTTGGCC
Salmon_SelPb2 1306 TCTGATAAATAGTTTTGTGTGAATTCAGTTCGATTTTGTCTGTGCCTGTCAATTGTATCTGATAAAGCAAACCTGTACAGTAAACTGGAGTCATTTGGCC

Trout_SelPb1 1044 CCTTGAAA-ACAGTATGTCTTCCCTACATGTG----GTCTGGTGACAGGTCCTGCTCTCCTCTGCAGTTTATGAAGTCTGCAGGTCAAACTCTACGAAGG
Salmon_SelPb1 1178 CCTTGAAATACAGTATGTCTTCCCTACATGTG----GTCTGGTGACAGGTCCTGCTCTCCTCTGCAGTTTATGAAGTCTGCAGGTCAAACCTTACGGAGG
Trout_SelPb2 1054 CTTGGAAT-ACAGTATGTCTTCCCTACATGTGACATGTCGGGTGACATGTTCTACTCTCTGC-------TATGAAGTCTGCAGGGCAAACCTTACTGAGG
Salmon_SelPb2 1406 CTTGGAAT-ACAG-------TCCCTACATGTG----GTCGGGTGACATGTTCTACTCTCTGC-------TATGAAGTCTGCAGGGCAAACCTTACTGAGG

Trout_SelPb1 1139 TGTCTGTAGATAGATGCTGTGCTAAGGAAAGTTGGACTGACAGTCACCGAACCCATGCAGGGCAGTGTGCTGGACTTCAGGGTAT
Salmon_SelPb1 1274 TGTCTGTAGAAAGATGCTGTGCTAAGGAAAGTTGGACTGACAGTCACCGAACCCATGCAGGGCAGTGTGCTGGACTTCAGGGTAT
Trout_SelPb2 1146 TGTCTGTAGACAGATGCTGTGCTGAGGAAAACGGGACTGACAGTCACTGAACCCATGCAGGGCAGGGTGCTGGATGTCAGGGTAG
Salmon_SelPb2 1487 TGTCTGTAGACTGATGCTGTGCTGAGGAAAACGGGACTGACAGTCACTGAACCCATGCAGGGCAGTGTGCTGGACTTCAGGGTAT
**B.**

**
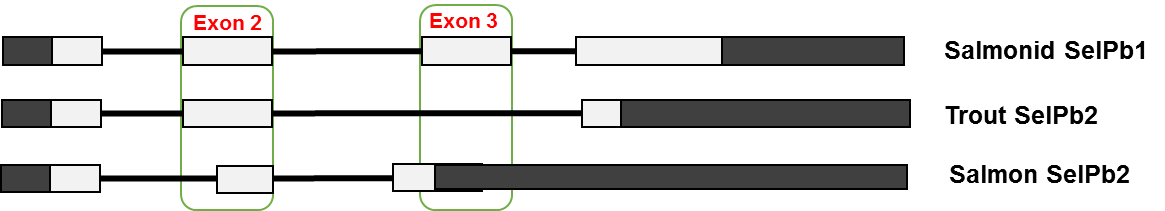
**

**S6 Figure: cDNA sequence alignment of salmonid SelPb paralogues (A) and schematic gene organisation of the coding region (B). A.** The accession numbers for trout and salmon SelPb1 are HF969250 and XM_014140446, and SelPb2 are MH085056 and MH085057, respectively. The start and stop codons of each main ORF are in red. The intron positions are indicated by red arrowheads. **B.** The intron/exon structure was predicted as in Figure 2. The coding regions are indicated by grey boxes and introns by black bars.
