# Supplementary material for: Four selenoprotein P genes exist in salmonids: Analysis of their origin and expression following Se supplementation and bacterial infection
Source: PLoS One. 2018 Dec 20;13(12):e0209381. doi: 10.1371/journal.pone.0209381 (PMC6301783; doi:10.1371/journal.pone.0209381)
Supplement: S7 Fig — (DOCX) [file pone.0209381.s007.docx]

S7 Figure

Human_SelP 1 --------------------MWRS------------------------------LGLALALCLLPSG---------GTESQDQSSL-CKQ
Rat_SelP 1 --------------------MWRS------------------------------LGLALALCLLPYG---------GAESQGQSPA-CKQ
Mouse_SelP 1 --------------------MWRS------------------------------LGLALALCLLPYG---------GAESQGQSSA-CYK
Platypus_SelPa 1 --------------------MWQG------------------------------LGLALALCLLPGG---------GAESQSASSH-CKE
Chicken_SelPa 1 --------------------MWAG------------------------------LGLALVLCLLPGG---------GTESQR-----CQE
Cocodile_SelPa 1 --------------------MWAG------------------------------LGLALVLCLLPEG---------GTESQGSSAR-CKE
Latimeria_SelPa 1 --------------------MWTG------------------------------LGLLLALCVLSRG---------RAESQGKSTR-CKE
Frog_SelPa 1 --------------------MWKG------------------------------FALALALCLLPWG---------GAESQGHRSR-CKQ
Pike_SelPa 1 --------------------MWAG------------------------------LSLLLALCLLPGG---------GTESEGEGSR-CKP
Trout_SelPa1 1 -------------------MMWVG------------------------------LSLLLALCLLPGG---------GTESEGEGTR-CKQ
Salmon_SelPa1 1 --------------------MWAG------------------------------LSLLLALCLLPGG---------GTESEGEGTR-CKP
Trout_SelPa2 1 --------------------MKAG------------------------------LSLLLALCLLPGG---------GAESEGEGTR-CKP
Salmon_SelPa2 1 --------------------MKAG------------------------------LSLLLALCLLPGG---------GAESEGEGTR-CKP
Stickleback_Sel 1 --------------------MWAY------------------------------LSLLVALCLLRGG---------GAESVGDGPH-CQL
Tilapia_SelPa 1 --------------------MWAG------------------------------LSLLLTLCLLHGG---------GAESEGGGPR-CQL
Medaka_SelPa 1 --------------------MWAG------------------------------LTLLLALCLLHGG---------GAESEGGGPR-CQP
Fugu_SelPa 1 ------------------MLVFAGGVAAVASEQRTFLPISULGESGGTAEMRACLGLLLALCMLHGG---------GAESEGDGPR-CQL
Zebrafish_SelPa 1 --------------------MWKA------------------------------LSLTLALCLLVGC---------SAESETEGAR-CKL
S-gar_SelPa 1 --------------------MWTG------------------------------LSLVLAVALLPVG---------GAESEGGGGR-CKT
Pike_SelPb 1 ---------------------MQG------------------------------FYLTLWLCATLSGPLQAS--PLFVEGDRNASRICKP
Trout_SelPb1 1 --------------------MMQG------------------------------L-LTLWLCAALPGLLCAS--PLLVEGDNDASKICKP
Salmon_SelPb1 1 --------------------MMQG------------------------------L-LTLWLCAALPGLLCAS--PLLVEGDNDASKICKT
Trout_SelPb2 1 --------------------MMQG------------------------------L-FTLRLCAALPGLLWAS--PLLVEGDNDASKICKP
Salmon_SelPb2 1 --------------------MMQG------------------------------L-FTLRLCAALPGLLWAS--PLLVEGDTDASKICKP
Stickleback_Sel 1 ------------------------------------------------------MSAVSLLYAALPGLLWASHVSLLVEGDNDASRICKP
Tilapia_SelPb 1 ---------------------MSS------------------------------L-LTLWLFAALPGLLWASHSTLIVEGDNDASRICKP
Fugu_SelPb 1 --------------------MSSH--------------------------------SLLWLQATLIGLLWAS----HVQGFNHTTRICKP
Medaka_SelPb 1 ---------------------------------------------------------MLRLCSALPALLWASLSVLSAEGDSNASKICKP
Zebrafish_SelPb 1 -----------------MQALWPL--------------------------------LLSALPALLGA------SSLFVEKESNGSRICKP
S-gar_SelPb 1 -----------------MRGLWAL--------------------------------LLTALPALQGA-------AAVAAQERNSSRLCQP
E-shark_SelP 1 ----------MIIKLCCRVVEAVV------------------------------MELGVPLLTVLLGL------VLIVSGEKQESRICQA
Whaleshark_SelP 1 -----------------------M------------------------------MELEMPLLTIVLGL------TISVVAAESESRICKV
Latimeria_SelPb 1 -----------------MMGRWAP--------------------------------VFTLLAGLVTG-----------FSPGNGSQICQP
Chicken_SelPb 1 --------------------MGP-------------------------------LLLALASCLGLAV---------ASEGATNGSRLCHE
Crocodile_SelPb 1 ------------------------------------------------------MGPPALAAAALLGLV-------AAALADVADRICQP
Frog_SelPb 1 --------------------MHKS------------------------------VLMISALMGLLGL---------VSSSEQTNSSICKP
Platypus_SelPb 1 ---------------------------------------------------------TLATLLAAAG---------ALPDLENGTRICQP
Whaleshark_SelP 1 --------------------MRKG------------------------------LGVALTLCLLLVG---------WVEGQEH----CNK
E-shark_SelP 1 -----------------------------------------------------------------------------------MSNDCQD
Amphioxus_SelPa 1 MSMGDEPLLFAATNSLSNMIHHRG------------------------------NLLAAALCLVVSG----------LDALAAPNRLCTQ
Amphioxus_SelPb 1 ---------------------MSG------------------------------LLQTAALCLVVS----------WLGSLTCAQEMCGP


Human_SelP 31 PPAWSIRDQ-DPMLNSNGSVTVVALLQASUYLCIIEASK----------------LEDLRVKLKKEGYSNISYIVVNHQGISSRLKYTHL
Rat_SelP 31 APPWNIGDQ-NPMLNSEGTVTVVALLQASUYLCLLQASR----------------LEDLRIKLENQGYFNISYIVVNHQGSPSQLKHAHL
Mouse_SelP 31 APEWYIGDQ-NPMLNSEGKVTVVALLQASUYLCLLQASR----------------LEDLRIKLESQGYFNISYIVVNHQGSPSQLKHSHL
Platypus_SelPa 31 APRWQIRDQ-DPMLNSLGTVTVVALLQASUYLCILQASR----------------LEDLRVKLENEGYSNISYIIVNHQGMPSQLNHKTL
Chicken_SelPa 27 PPEWHIGEE-SPMLNARGSVAVVALLQASUYLCLLQASR----------------LEDLRVKLENEGLVNISYVVVNHQSPHSQKKFHLL
Cocodile_SelPa 31 PPEWYIAGQ-DPMLNSRGSVTVVALLQASUHLCLLQASS----------------LEDLRVKLENDGLVNISYVVVNHQGITSQEKIHLL
Latimeria_SelPa 31 PPTWYIGEE-NPMLESRGKVTVVALLQASUHFCLMQASR----------------MDDLHLKLENEGLVNISYMIVNHQGEYSQHKHNLL
Frog_SelPa 31 PPDWSIGDQ-NPMIQSAGKVTVVALLQASUYLCLLQASR----------------LEDLRLKLEKEKLVGISYVVVNHQGRHSRAKYDLL
Pike_SelPa 31 PPIWSIGEV-EPMKEAMGQVTVVALLQASULFCLVQASL----------------LDGLRLKLEGQGLKNVHYMVVNHQGEKAQRLHKLL
Trout_SelPa1 32 PPGWSIGEV-EPMKEVMGQVTVVALLQASULFCLVQASL----------------LDGLRLKLEGQGLENVTYMVVNHQGEQAQRLHTLL
Salmon_SelPa1 31 PPGWSIGEV-EPMKEVMGQVTVVALLQASULFCLVQASL----------------LDGLRLKLEGQGLENVTYMVVNHQGEQAQRLHTLL
Trout_SelPa2 31 PPGWSIGEV-EPMKGVMGQVTVVALLQASUSFCLVQASL----------------LDELRLKLEGQGLDNVTYMVVNHQGEQAQHLHTLL
Salmon_SelPa2 31 PAGWSIGEV-EPMKGVMGQVTVVALLQASULFCLVQASL----------------LDELRLKLEGQGLDNVTYMVVNHQGDQAQHLHTLL
Stickleback_Sel 31 PSPWRIGEV-EPMQGTMGRVTVVALLQASULFCLVQASR----------------MDGLRQKLESQGLKDVAYMVVNQQGEQARRLHPML
Tilapia_SelPa 31 PSDWRIGDV-EPMKGSVGRVTVVALLQASULFCLVQASR----------------LDGLQQKLERQGLKNVVYMVVNHQGEQSRHLHPLL
Medaka_SelPa 31 PPAWKIGEV-DPMKESMGRVTVVALLKASULYCLVQASR----------------LDGLHKKLEGQGLKDVVYMVVNHHEAQAQRLHPLL
Fugu_SelPa 63 PPVWKIGDL-EPMKEAMGRVTVVALLESSULFCVVQASR----------------MDSLRQRLENQGLRDVVYMVVSHQGAHAPGLHAML
Zebrafish_SelPa 31 PPEWKVGDV-EPMKNALGQVTVVAYLQASULFCLEQASK----------------LNDLLLKLENQGYPNIAYMVVNNREERSQRLHHLL
S-gar_SelPa 31 PPEWAVGNE-QPMTDSAGRVTVVALLQASULFCLVQASR----------------LDVLRLRLEQQGLVNISYVVVNHQGEQSHRLYPVL
Pike_SelPb 38 APAWEIKGQGAPMKELLGNVVVLALLKASUHFCLTQASK----------------LQGLREKLLRGNLTDVSFLIVNEREAQSRAMYWEL
Trout_SelPb1 38 APHWEIKGHGAPMKELLGNVVVLALLKASUHFCRTQASK----------------LGGLRDKLLRSNLTDVSFLIVNEREAQSRAMYWEL
Salmon_SelPb1 38 APHWEIKGHGAPMKELLGNVVVLALLKASUHFCRTQASK----------------LGGLRDKLLHSNLTDVSFLIVNEREAQSRAMYWEL
Trout_SelPb2 38 APRCEIKGHGAPMKGLLGNLVVLALLKACUHFCLTQASK----------------LEGLHDKLLRSNLTDMSFLIVNEREVQSUAMYWEL
Salmon_SelPb2 38 APRWEIKGHGAPMKGLLGNVVVLALLKASUHFCLTQASK----------------------------RSSPEPCIVNURGAPPP------
Stickleback_Sel 37 APHWTIKER-APMQELLGNVVVVALLKASUQFCLTQASK----------------IGGLRDKLNRSNLTDVSFMIVNEREPHSRAMYWEL
Tilapia_SelPb 39 APQWDIKGY-APMQDLLGNVAVVALLKATUQFCLKQASK----------------IGGLRDRLNRSNMTEVSFIIVNERDAHSRAMYWEL
Fugu_SelPb 35 APHWEINGE-APMQRLLGRVAVVALLKATUHFCLVQASR----------------IGGLRKKLIQSNMTEVSYMIVNEQDPHSRALFWEL
Medaka_SelPb 34 APYWDIEGH-VPMQEHLGNVVVVALLKATUEFCLTQASK----------------IGNLRDKLNRNNITEVSFMIVNELEALSQTMHWKL
Zebrafish_SelPb 36 APQWEIDGK-TPMKELLGNVVVVALLKASUHFCLTQAAR----------------LGDLRDKLANGGLTNISFMVVNEQDSQSRAMYWEL
S-gar_SelPb 35 PPRWEVSGR-APMEELRGRVAVVALLKASUQFCLTQASR----------------LGDLRTRLSRSGMTDVGFLIVNEREPVSRAMFWEL
E-shark_SelP 45 APLWEIANQ-NPMEQQLGSVVVVALLKASUQFCLTQAAK----------------LGSLQDKLARQGLKDVHYMIVNEKAPESRAMLWEL
Whaleshark_SelP 32 APHWEIADR-KPMEEQIGHVVVVALLKASCGFCLTQATK----------------LGSLRDKLKHQGLKDIHYMIVNEKSPQSRAMLWEL
Latimeria_SelPb 31 APVWEINGE-RPMEETLGRVTVVGLLEASUHFCLMQAAR----------------LGSLHEKFARKGMEDIRYLIVNNKSPSSRAMFWEL
Chicken_SelPb 31 APAWRINGS-SPMEGAAGQVTVVALLKASUHFCLLQARS----------------LGALRERLGQQGVSDVRYVIVNEQAPLSRAMFGEL
Crocodile_SelPb 30 APLWRVNGT-VPMDEALGQVTLVALLKASUQFCLKQASS----------------LGGLREKLSRRGMADVSYMIVNEKAPLSRAMYWEL
Frog_SelPb 32 SPKWSIDGE-VPMAEALGNVTVVALLQASCGFCLIQAAR----------------MGPLRDKLYLQGMTDIKYLIVNDQSKTSTDMFPEL
Platypus_SelPb 25 APRWTVNGV-APMEGTEGQVIVVALLKASUHFCLKQAARRRGCPFLGPLPVEGGGLAGLRERLAGHGAGNVSFLIVNQRDPTAQLLHTEL
Whaleshark_SelP 28 PPAWTIGGK-EPMTDSLGKVTVVALLQASUHFCLTQAAS----------------LTVLQQKLHENGFVNVSFIVVNHQGKSSREKYQHL
E-shark_SelP 8 ------GGR----------------------------------------------LDSLRKNLTNAGLVNISYLIVNHQEKLSRDLYQNF
Amphioxus_SelPa 51 PPSWELDGI-DFMEESRGNVVVLQMSIAIUSFCRTQAER----------------LERLRLKLQRDGVTDISFGVINGMGFMSRVSLRQL
Amphioxus_SelPb 30 PAYWEAEGR-SPMADNKGKVIVMKFPIGSUPFCQGEVSS----------------LEALRQKLYSEGKSDIFFGAVNHWGWASWWYRGEL

S7 Figure, continued

Human_SelP 104 KNKVSEHIPVYQQEENQTDVWTLLNGSKDDFLIYDRCGRLVYHLGLPFSFLTFPYVEEAIKIAYCEKKCGNCSLTTLKD--------EDF
Rat_SelP 104 KKQVSDHIAVYRQDEHQTDVWTLLNGNKDDFLIYDRCGRLVYHLGLPYSFLTFPYVEEAIKIAYCEKRCGNCSFTSLED--------EAF
Mouse_SelP 104 KKQVSEHIAVYRQEEDGIDVWTLLNGNKDDFLIYDRCGRLVYHLGLPYSFLTFPYVEEAIKIAYCEERCGNCNLTSLED--------EDF
Platypus_SelPa 104 KEKVSEHIPVYQQDEKQTDVWSTLKGNKDDFLIYDRCGRLVYHLSLPYTFLSFSYVEDSIKTTYCEQNCGNCSYTMPEA--------EEF
Chicken_SelPa 100 QESVSDHITVYQQDDHQADVWTTLNGNKDDFLIYDRCGRLVYHLGLPYSFLSFQYVEEAIKIAYCENNCGNCSYTEPDI--------DNI
Cocodile_SelPa 104 RKEVSEHIVVYQQDENQPDVWTTLNGNKDDFLIYDRCGRLVYHLGLPYSFLSFLYVEESIKIAYCEQNCGNCSYMTPDV--------EKV
Latimeria_SelPa 104 KQRVSEFIPVYKQDMEKPNVWNLLNGNKDDFLIYDRCGRLVYHLGLPYSVLHFPYMEEAIRIAFCETRCGNCSFTPEDI--------NEI
Frog_SelPa 104 KSKVSEHIPVYQQEENQPDVWSLLKGDKDDFFIYDRCGRLVQHLELPYSLLHFPYVEEAVRIAYCGDKCGECEHKIPDA---------DV
Pike_SelPa 104 KQKLSEKITLYKQEPEQVDVWQSLAGQKDDFLIYDRCGRLTYHISLPYSIMSIPYVENAIKETYCARICGNCKHESVEI--------PAE
Trout_SelPa1 105 RQKLSENITLYKQQPKQEDVWQTLAGEKDDFLIYDRCGRLTYHISLPYSILGTPYVENAIKETYCTRVCGDCTYESKEI--------PAE
Salmon_SelPa1 104 RQKLSENITLYKQQPKQDDVWQTLAGEKDDFLVYDRCGRLTYHISLPYSILGTPYVENAIKKTYCTRICGDCSYESQEI--------PAE
Trout_SelPa2 104 SQKLSENIILYKQVPKQDDVWQALAGKKDDFLIYDRCGRLTHHIFLPFSILGTPYVENAIKETYCQRICGDCTYENTEI--------PAE
Salmon_SelPa2 104 SQKLSENIILYKQEPKRADVWQALAGKKDDFLIYDRCGRLTHHIFLPFSILGTPYVENAIKETYCQSICGDCTYESTEI--------PAE
Stickleback_Sel 104 AQRLSVNIDLYKQDEQQPDVWKTLGGDKDDFLVYDRCGRLTHHIALPYSIIGQGHVESAIKDAYCKRTCGDCVHESATT--------PEE
Tilapia_SelPa 104 EAKLSKNIILYKQDGHQPDVWQTLAGEKDDFFIYDRCGRLTYRISLPYSIIGEGHIEKAIKDTYCKRLCGDCTHESAEI--------PEE
Medaka_SelPa 104 EERLSQNIALYKQYEHQPDVWRTLNGEKDDFFIYDRCGRLTHHLSLPYNIIGHGHVEQAIKEAYCNRVCGECSFESADT--------PAE
Fugu_SelPa 136 AQKLTEHISLYKQDEALPDVWQTLGGNNNDFFIYDRCGRLTHRISLPYSIIGHGHVERAVKDTYCNSLCGECTHETTET--------LQE
Zebrafish_SelPa 104 QERLL-NITLYAQDLSQPDAWQAVNAEKDDILVYDRCGRLTYHLSLPYTILSHPHVEEAIKHTYCDRICGECSLESSAQ--------LEE
S-gar_SelPa 104 KKKMSQKIRVYDQDPLQEDVWKILSGEKDDFLIYDRCGSLTHHLGLPYSMLTMSYVEDAIRDTYCKDICGNCSLESSSL--------PVT
Pike_SelPb 112 RRRASPAIPVYQQAPLQDDVWEALDGDKDDFLVYDRCGRLTFHIVLPYSFLHYPYIEAAIRATYHKDICGNCTLDFNST-----------
Trout_SelPb1 112 KRRAPPGIPVYQQAPLQDDVWEALDGDKDDFLVYDRCGRLTFHIVLPYSFLHYPYIEAAVRATYHKDICGNCTVDSNTT--------SSA
Salmon_SelPb1 112 KRRAPPGIPVYQQAPLQDDVWEALDGDKDDFLVYDRCGRLTFHIVLPYSFLHYPYIEAAVRATYHKDICGNCTVDSNTT--------SSA
Trout_SelPb2 112 KRRATPDIPVYQQAQLQDDVWEALYGNKDDFLVYDRNPNGVTQLG------GTADSETSLU----------AAQEWLLT------RQTLQ
Salmon_SelPb2 94 ----PPGIPVYQQAQLY--------GNKDDFLVYDR------------------------------DLRNWCD--------------TSF
Stickleback_Sel 110 KRRAPPGVPVYQQAALQSDVWEALDGDKDDFLVYDRCGLLTFHIVLPYSFLHNVYVEAAIRATYLKNIC-NCTVDSVVSSLNNSVMNNET
Tilapia_SelPb 112 KRSAPTGVPVFQQQPFQNDVWEALDGDKDDFLVYDRCGLLTFHIVMPYSFLHHPFVEAAIRATYQKNIC-NCTQNFTSS--------VGG
Fugu_SelPb 108 ERRAPPDVPVYQQSAFQSDVWETLDGDKDDFLIYDRCGQLTFHVGLPYSFLNYVYVEAAIRATYQGNIC-NCSANSTSL-------HDTG
Medaka_SelPb 107 KKKAPTGVPVYQQSSLQKDVWEILDGDKDDFLIYDRCGLLTFHIVLPNSFLQNADVENAITATYTQDIC-NCSGNSTLS---------GG
Zebrafish_SelPb 109 KRRTAQDIPVYQQSPLQNDVWEILEGDKDDFLVYDRCGYLTFHIVLPFSFLHYPYIEAAIRATYHKNMC-NCSLNANFS--------ISE
S-gar_SelPb 108 KRRAPEGIPVYQQGLFQSDVWEILEGEKDDFLIYDRCGLLTFHIVLPYSFLHYPYVEAAIRATYLRDICGNCTLDSSDNQGLNASQIPER
E-shark_SelP 118 KRHVPNNVSVYQQSPIQPDVWHSLQGGKDDFLIYDRCGRLTFHVVLPYSSLQYPYIEAAIRATHKRDICGECTITKSSL----------E
Whaleshark_SelP 105 KRHTPEDIPVYQQSPFQRDVWSILQGNKDDFLIYDRCGKLTFHIVLPYSYLQFRYTEAAIRATYNKDICGNCGIGNISL----------E
Latimeria_SelPb 104 KRHAPANITVYQQAPLQKNVWQILEGKKDDFFIYDRCGKLTFQISLPFSFLNNSYVEAAIMSTYHSDKCGNCSAANFNQ--------TVQ
Chicken_SelPb 104 QRHAPPGVPVLQQQPHEPDVWQLLGGDKDDFLVYDRCGRLAFHIQLPYSFLHLPYVESAIRFTHRKDFCGNCSLYPNST---------QE
Crocodile_SelPb 103 KRQAPAGVPVYQQGVLDPDVWQILDGDKDDFLIYDKCGRLVFHISLPYSFLHFPYVESAVHFVYHKDYCGNCFHYSNST--------QQE
Frog_SelPb 105 KRWAPKGIPVYQQTPGQEDVWDLLNGNKDDFLIYDRCGRLTFHIRLPLSFLHFPYVEAAIKFTYNESFCGNCSFTSNST--------LMP
Platypus_SelPb 114 ERHAPPGVPVYAQDGPDPDVWSVLGGDKDDFFVYDRCGRLTFHIQLPFSFLHFPYVEAAVRFTHRRDFCGNCSYYFPQV-----------
Whaleshark_SelP 101 NSKVENQIPVYQQDVNQPDVWTLLRGIKDDFLIYDSCGHLTYHLELPYTILSQPFVAHAIVKTYCQRICSNCSFVQDNS--------PAC
E-shark_SelP 46 TDLVSRDIPVYQQNVHQPNIWKLLQGVTDDFLIYDRCGHLTYHLGLPYTLLTLPYVETAIRMTYCQNICSGCSVMQY----------VEA
Amphioxus_SelPa 124 EGVV--NFGVYQDTP-RADVWGLLDGRKDDFIIYDRCGRLARHIRMPEAWLVRPDVEDAIREVYAESPCGNCAFYPDTT--------PTP
Amphioxus_SelPb 103 ERRA--NFPVY-QDSWSQDIWGKLHAAKDDILVYDRCGRLAYHLRLPRAYLGNTHTEEAIRAAYRQSPCGPCGT----------------


Human_SelP 186 CKRVSLATVDKTVETPS----------PHYHHEHHHN------------HGHQHLGSSELSENQQPGAPNAPTHPAPPGLH---------
Rat_SelP 186 CKNVSSATASKTTEPSE----------EHNHHKHHDK------------HGHEHLGSSKPSENQQPGALDVETSLPPSGLHHHH------
Mouse_SelP 186 CKTVTSATANKTAEPSE----------AHSHHKHHNK------------HGQEHLGSSKPSENQQPGP--SETTLPPSGL---H------
Platypus_SelPa 186 CTNTSSAAKEKATEAPL-------PHNDRPHHHHHHH------------HHHGHKPHPSGTEQAPADPDGPLRSPAPQGLH---------
Chicken_SelPa 182 CENITKKEDENLAGIEP-------EPEPSGQHSHHHH--------QLHRHRHHHHHREGGRHSKTQNHQAPSESQRRHPHNG--------
Cocodile_SelPa 186 CNNIDKSTDEEPAEITP------EQHNHHSHQPNQHR-----------HHGHGHHHREEDQLSEDQNQQAHAQRHHSPGNNR--------
Latimeria_SelPa 186 CNRTENTDEPVEEPPPT------SVPDPPQQNPQHHG----------AHRHHRQHGHQEQSHHHRHDQFSSGQNGQVHV-----------
Frog_SelPa 185 CKKPEEQPEQEKPVEEK-----VERPRPHRNHHRHHR-------PKHSGHRHRHHNNEGGQAAEVDAFQTNNRAGSHNG-----------
Pike_SelPa 186 CNGTAEATSEGEDKPTT---TVEPTHDGHQHHRHHHH-----------HHHHDDKHGDHGDREVGRGHGAEQQRHHKHVGEGHRHV----
Trout_SelPa1 187 CNRTVEAKPEGEEKPVT---GRETTHGGHGHHHHGHGHNGNRHGHNGNRHGHDHHGERGMGRGHGRDHGAER--QHQHDTEGLQHGQA--
Salmon_SelPa1 186 CNRTVEAKPEGEEKPVT---GRETTHGGHGHHHHGHGHNDNRHGHNGNRHGHDHHGERGMGRGHGRDHGAERQQQHQHDTEGLQHGQA--
Trout_SelPa2 186 CNRMVEVKPEGEEKPVT---GGDTPHGGRGHHHHGNG-----HGHHGKSHGHGHHGESDVGREHGRGHGVEQ-QQHQNGAERLHHGQA--
Salmon_SelPa2 186 CNRMVEVKPEGEEKPVT---GGDTPHGGRGHHHHGNG-----HGHHSKSHGHGHHGESEVGRDHGRGHGVEQ-QQHQHGAEGLHHGQA--
Stickleback_Sel 186 CVEKAVAQPDADAPPVVEDNGGGGGHHGHHHNGHGHH----RGHHHGHHHGHHHGGDHGVGQQAVVHQEHERDGGASHGQHNSALDQM--
Tilapia_SelPa 186 CKDNAGVQPDVPAEQDD---TRHDHHHGHGHGHHHGH---GH--HHG--HGHGHHGDNQDVHPH--GHGSDHNNGHHHRNHDGADQTQHG
Medaka_SelPa 186 CQGKDNAQPETDGAQPA--------GEEHQHQHPHHG------------HHGHNHGDNHGLHPRGFGHGHDHRHGHHHRHHHGRA-----
Fugu_SelPa 218 CTPKTSALPDNGVAPGA-------EETGHECHHHGRQ-----------HHGDGHRDHGDSQCTHTRGSGRGHGHHHGHGGQGQH------
Zebrafish_SelPa 185 CKKATEEVNKPVEEEPR----------------QDHG-----------HHEHGHHEHQGEAERHRHGHHHPHHHHHHH------------
S-gar_SelPa 186 CNSTVKPEGKPEDKPEE--------SPESHGHPHAHG------------HGHHHSEEGRQSHRRHSSHGHHHGHGRHHHGSQQEFNNG--
Pike_SelPb 191 TWNSTQHKSLSGVGVIV---------------NETDG------------TAASALTVTENSSPAPNEGGVTQTHHHGPG-----------
Trout_SelPb1 194 GWNSTRRNETLSSSEMRVNETDSTVRSIDVDTVSNPV----------PSDGPQMSAEGGGNMSHIHHQHHQYPHH---------------
Salmon_SelPb1 194 GWNSSQRNETLSSSEMRVNETDSTVVDTVRSTDVDTV------SNPVASDGRQMSSEGGGNMSHIPHQHHQYPHHQHHQYPH--------
Trout_SelPb2 180 CQIEVATVSNPVPDVVU-------------------------------------------------------------------------
Salmon_SelPb2 128 MWPWISLLSLIDV-----------------------------------------------------------------------------
Stickleback_Sel 199 DFNVSQTNATPRIQPDTNDPEGAGTPPPPTHHHSHHH------------HHHHQHHHPHHPHLNQPLHDTSHPLSVHH------------
Tilapia_SelPb 193 NNITNNGTAQLVDEGSEVPDTASGRTHHHHHHHHHHD------DPPSHHHHHDQHSSHQNQSLHQQDQGDSTSHHHHHS-----------
Fugu_SelPb 190 RNETAQALTQLGADDPQVTPAPVRLHHHHHHHHHHHH------------HRHDHPHQDGSSKPSTPTP----------------------
Medaka_SelPb 187 GNNFTRNSSQSHSGVQRPADEPENVTTTLEASPNDSR----------HEHAHHHHPHHQHLHHHHPHHQHLHHHHPHHQHLH--------
Zebrafish_SelPb 190 SPDSTKNEPAGENNQRP----NSTEPVTAAHHHHHQQ-------HEPHHHHHNPYPNSHKKSGDSDVTGKPKEPTHH-------------
S-gar_SelPb 198 KENVTDAECLDDHEQHQGEQSNGPQPSRHSQAHHSGH--------RLVHGPCRDKGRKRPPSPQNSTLSARQHAHSPHTI----------
E-shark_SelP 198 MDRNVTTTPSRHLSPNT-------------------------------------------------------------------------
Whaleshark_SelP 185 TKRNITSYKANIFQFNTTSFVASLTFENITTQPVQQH----------------------VANKPDPDPKLSSSPIHPH------------
Latimeria_SelPb 186 VGSNTSVSAWEESGK----------------------------------EGLGQNKQSVEDYIQHPLARLDPLPSLPP------------
Chicken_SelPb 185 ANSTMEVPATLTPLPKQ--------EEKESETPAHHQ------------PNHLHPHHRAVGNGTAPEPSGDHRPAHAHH-----------
Crocodile_SelPb 185 ANGTAEAQANPSLAPRQ-------------------------------------EKEEEPPPRVHPTPRHEADGSEASVLHG--------
Frog_SelPb 187 MNETALLSLSDNSSSPL--------------------------------------PNKDGPVNKEPSKTLEKNKDHKKLDSDR-------
Platypus_SelPb 193 -NDTTTQESELEKSPGA------------------------------------PGEEPEGSPVREPDRPQSQDPTGPF------------
Whaleshark_SelP 183 IARNITQTLKIDLRKHH-----------RGHHHAHRN------------HNPNVHEEDQLQQNRTDPVQLGHNTHKHHQ-----------
E-shark_SelP 126 CNSTSATEENTADKTVQNHRRGVVKSVNKADAQPDSG---------LNTHTQLLLNMTGPGEEQDPHRRRQHRHHQRHHK----------
Amphioxus_SelPa 203 ILTTTPGSPTDGPDVTTASDIGSGDLDEGFIRDTGTSMDDVDESNAPLHTSTSHQNRTSVNNRTERIMFTEHNTESPQRNSKRHHRR---
Amphioxus_SelPb ------------------------------------------------------------------------------------------
S7 Figure, continued

Human_SelP 245 ---HHHKHKGQHRQGHPENRDMPASEDLQD------------LQKKLCRKRCINQLLCKLPTDSELAP---RSUCCHCRHLIFEKTGS--
Rat_SelP 248 ---HHHKHKGQHRQGHLESUDMGASEGLQLSL----------AQRKLURRGCINQLLCKLSEESGAAT---SSCCCHCRHLIFEKSGS--
Mouse_SelP 243 ---HHHRHRGQHRQGHLESUDTTASEGLHLSL----------AQRKLURRGCINQLLCKLSKESEAAP---SSCCCHCRHLIFEKSGS--
Platypus_SelPa 248 ---KRLRPAGQPRQGQGGSREAAEGRGEELPSPRK-------KAURKGNASCQNQLLUDWHKRSGPAP---SSUCUHCRHLLFGSKATA-
Chicken_SelPa 249 ---RRHRVFNHNRHDQIGSHEQVETLPPGEGVENLPRVT---KLUKKGKTICKNQLTUNWQTASDSTT---SSUCCHCRHLLFEELGN--
Cocodile_SelPa 251 ---RHNRVLGRNRQDQAGSQERVEAVPQREVLEIPLQSK---RLUKKGNASCKNQLTUNFLKASESTS---SSUCUHURHLLFEEVGK--
Latimeria_SelPa 249 ---EVQRPIAHDHHGNEQGQVDSLLIRQVR------------RPUKKKAASUSQSVEUNWQEMSGLIS---SKUCUHURQLYSNQAIDN-
Frog_SelPa 252 ---QGQSVVPQSEVVFVPQREADVPVLA--------------LQPUKKAKSUKKQYLUEWREDAGKAF---NSUCUHURQLSFEIAQN--
Pike_SelPa 258 ---QDQLHVSQDHVGQTAVQL---GQETNEGQVMQ-------RPUAKEGVRCSGQHDUQWKEGSVLSPSSKASUCUHURHLLDDGVSGQP
Trout_SelPa1 270 ---HGQLHVGQEHMGQQAVQLGQMPQEGQRGHIMQ-------NPUVKGKSRUKAEHSUQWKEGSDLSPSSKASUCUHURRLFGDGVSNEP
Salmon_SelPa1 271 ---QGQLHVGQEHMGQQAVQLGQMPQEGQRGHIMQ-------NPUVKGMSRUKAEHSUQWKEGSDLSPSSKASUCUHURRLFGDGESNEP
Trout_SelPa2 265 ---HGQVHVGQEHMGQQ-------PKEVQEGHIMQ-------RPUVKGRARUKAELSUHLKEGSDISPSSKVSUCUHURGLFGNGVRNEP
Salmon_SelPa2 265 ---HGQLHVGQEHMGQQ-------PKEAQEGHIMQ-------RPUVKGRARUKAELSUHLKEGSDISPSSKVSUCUHURGLFGNGVSNEP
Stickleback_Sel 270 ---QQQAHIPQMPHGAQAAPV---------------------RPUVEENAKUKSKHSUKLTAGSDNEASLKLSUCCHURRLFGEVGSEQP
Tilapia_SelPa 264 VRPHGHFHEGDMPQTQHHFDLGQIPQEVHNQQVAQEAHAVIERPULSRKNRUKLKYNUQGLTGSDNEI--KSSUCUHURRLFGEAGSEQP
Medaka_SelPa 251 ---ETRLQEHQHHASSDQMQHAVQLEQIGQEVVGAPV-----RPUVQETARUKTKFTUHMVAGSENE----ASUCUHURRLFGHAGSEQP
Fugu_SelPa 284 ---EGRVHMGDIPQRPDHLDLGQAQQALNLHHLPQHDAA---ATRPUESKRUKAQFSUQWAEASDTGAFPKASUCUHURRLFGDVVGEEP
Zebrafish_SelPa 236 ---RGQQQVDVDQQVLSQVDFGQVAAETP-------------MMKRPUAKHSRUKVQYSUQQGADSPV---ASUCUHURQLFGGEGNGRV
S-gar_SelPa 254 ---QTQRHSQTHQQQQLIKNPSLLSQQEVVDFPLRIE-----WPUKKGAAKUKHQYSUEWKEGSSDLLS--SSUCUHCRQLFGGEGUDQ-
Pike_SelPb 243 ---QHHQNKHYQRPHTHGSDI---------------------------------------------------------------------
Trout_SelPb1 259 ---HQQQHQHHHNHGSDA------------------------------------------------------------------------
Salmon_SelPb1 270 ---HHHYHQHHUNHGSDA------------------------------------------------------------------------
Trout_SelPb2 197 ------------------------------------------------------------------------------------------
Salmon_SelPb2 141 ------------------------------------------------------------------------------------------
Stickleback_Sel 265 ---HHHPHHHHHQQQQPGNHS---------------------------------------------------------------------
Tilapia_SelPb 266 ---PHQQHQHDH------------------------------------------------------------------------------
Fugu_SelPb 246 ------------------------------------------------------------------------------------------
Medaka_SelPb 259 ---HHHPHLQQHSKHQNDDSY---------------------------------------------------------------------
Zebrafish_SelPb 256 ---SHQEHVHNHR-----------------------------------------------------------------------------
S-gar_SelPb 270 ---HSHHDLHQHUAGCDR------------------------PPRAELQRQCTFT----------------ATACSLCALL---------
E-shark_SelP 215 ------------------------------------------------------------------------------------------
Whaleshark_SelP 241 -------HFNNSQDGSLET-----------------------------------------------------------------------
Latimeria_SelPb 230 ---PPHRHLHKRHQRFPE------------------------------------------------------------------------
Chicken_SelPb 244 ---HHGAHGKLHPKGQT-------------------------------------------------------------------------
Crocodile_SelPb 230 ---KAGPAHGRHREG---------------------------------------------------------------------------
Frog_SelPb 232 ---RPHDHSQHQPLNSHTPQE---------------------------------------------------NQNYHPRNLIKTG-----
Platypus_SelPb 234 ---SGVLLQGKENK----------------------------------------------------------------------------
Whaleshark_SelP 239 ---NHHHPHHQYDQSNSESETPDMVAGNGS------------PQPUINKSNCKRKFKUKPEGMSELAG---IRUUUYCRQLFSDKSSNNE
E-shark_SelP 197 ---NHHNNSGHREESMAGTSDGPTANSPRVQRNQHSLQI---RPURVMKFKCIPEVRSEVAP---------VSUAUHURQLFGSDLSHNV
Amphioxus_SelPa 290 ---HHHRRHGDHRQEAHSESH---------------------------------------------------------------------
Amphioxus_SelPb ------------------------------------------------------------------------------------------


Human_SelP 315 AITUQCKENLPSLCSUQGLRAEEN--ITESCQURLP----PAAUQISQQLIPTEASASURUKNQAKK-----UEUPSN-----
Rat_SelP 320 AITUQCAENLPSLCSUQGLFAEEK--VIESCQCRSP----PAAUH-SQHVSPTEASPNUSUNNKTKK-----UKUNLN-----
Mouse_SelP 315 AIAUQCAENLPSLCSUQGLFAEEK--VTESCQCRSP----PAAUQ-NQPMNPMEANPNUSUDNQTRK-----UKUHSN-----
Platypus_SelPa 324 TALUQCRDALPALCSUQGRQSGED--VIESUQURSPLPAUPPAAQLPSPSPTDPNAAUKUENTAGM------UKUPTR-----
Chicken_SelPa 328 SITUQCRGALPNSCRUHGQLLAED--ITESUQURLL----TAAUESAAGGGSETSDTUQUQERAGN------UAUKTN-----
Cocodile_SelPa 330 TATUHCRGTLPDTCRUQGQLLAEN--ITESUQURLL----PAAUQSLAEREHETSDNUQUQGKTGN------UAUKTN-----
Latimeria_SelPa 320 AAAUQCKRMLPSAUQUQGQLNGIN--VFESUQURSP----RATUQPLSDRPSQQEAADSVAUKUSQNLEIUEUQHQEQ-----
Frog_SelPa 320 EVAURCQEALPASUQUQELLSDS---LSESUQURLS----AAAUHSHSTGLPELDTESETNAPUAUPQEAENUQUKELURFLM
Pike_SelPa 335 IRLUHCNEALPASCQUHGLKGL----LRETUQURSP----QTDUQQPRPV---MUAUSPGVES--------------------
Trout_SelPa1 350 IGLUHCDEALPASUQUQGLIG-----LRETUQURSS----LADUQQPQPV---MUAUPLGVES---------UGUGLL-----
Salmon_SelPa1 351 IGLUHCDEALPASUQUQGLMG-----LRETUQURSS----PADUQQPQPV---MUAUPLGVES---------UGUGQL-----
Trout_SelPa2 338 IGLUHCDEALPASUQUQGLMGDSNNHIRETUQURLP----PTDUQQPLPV---MSAUSPGVET---------USUEQL-----
Salmon_SelPa2 338 VGLUHCDEALPASUQUQGLMGDSTNHIRETUQURSP----PADUQQPPPV---MSAUSPGVET---------UGUEQL-----
Stickleback_Sel 336 LGLUHCDEALPASUQUHGLTDGVANNVRETUQCRSP----PAAUQQPEPAPUAUAAGVS-------------UGUEQL-----
Tilapia_SelPa 352 VGLUHCDEALPTSURUHGLIGDAVNDVRETUQURLP----HAAUQEPQPA---QUAUPPGVVS---------UGUEQL-----
Medaka_SelPa 329 LGLUHCQEELPASUQCRGLTGELANTVIESUQURSP----PAAUQQPQPA---QUAUAPGAAS---------UAUEQL-----
Fugu_SelPa 368 VGLUHCSEALPASUQURGPTGDAVNAVRETUQURSP----LAVUQQPQPAQUAUPQGVN-------------UGUEQV-----
Zebrafish_SelPa 307 AGLUHCDEPLPASUPUQGLKEQDNH-IKETUQURPA---PPAEUELSQPTUVUPAGDAT-------------UGURKK-----
S-gar_SelPa 333 AAFUHCEEKPPASCQUQGLQGGNH--VRETUQURSS----SKAURLPSAAGQPEQSUTUPQGNER-------UIUQEQ-----
Pike_SelPb 261 ------------------------------------------------------------------------RGLGSN-----
Trout_SelPb1 274 ------------------------------------------------------------------------DKQDSN-----
Salmon_SelPb1 285 ------------------------------------------------------------------------DKQDSN-----
Trout_SelPb2 197 ------------------------------------------------------------------------GGR--------
Salmon_SelPb2 141 ------------------------------------------------------------------------GDURAT-----
Stickleback_Sel 283 ------------------------------------------------------------------------GTAVDH-----
Tilapia_SelPb -----------------------------------------------------------------------------------
Fugu_SelPb 246 ------------------------------------------------------------------------EPKMAA-----
Medaka_SelPb -----------------------------------------------------------------------------------
Zebrafish_SelPb -----------------------------------------------------------------------------------
S-gar_SelPb 308 -------------CIYRGYC---------------------------------------------------------------
E-shark_SelP 215 ------------------------------------------------------------------------SD---------
Whaleshark_SelP 253 ------------------------------------------------------------------------DDTERL-----
Latimeria_SelPb 245 ------------------------------------------------------------------------AELVLG-----
Chicken_SelPb 258 ------------------------------------------------------------------------PEGRDP-----
Crocodile_SelPb 242 ------------------------------HQRRAPA-----------------------------------ARDEAR-----
Frog_SelPb 263 ------------------------------------------------------------------------AQRPEN-----
Platypus_SelPb 245 -----------------------------IIPWKTPLQAAPRKPSHP-------------------------PGAHD------
Whaleshark_SelP 311 VTUHCRTQSSSUQUQEQGRV--DS--LIESUQURLIPALUPQDQMSQRHUGPUQGVKSRE------------URDGLK-----
E-shark_ SelP 272 VTUQCRKTHLPSSUWUQQPKSEGN--VPEVUQURSL----SAASU---------------------------PENSQS-----
Amphioxus_SelPa 308 ------------------------------HHQRSQ------------------------------------DGHTDE-----
Amphioxus_SelPb -----------------------------------------------------------------------------------

**S7 Figure:** **Protein alignment of all selenoprotein P sequences used in the phylogenetic analysis.** All sequences were obtained from public databases and hand curated except the salmonid SelPa2 and SelPb2 sequences that were cloned in this work. Shading illustrates conserved regions in the alignment. The species accession numbers are Human SelP (CAA77836), Rat SelP (NP_062065), Mouse SelP (NP_033181), Trout SelPa1 (CCX35038), Salmon SelPa1 (XP_014026610), Trout SelPb1 (CCX35039), Salmon SelPb1 (XP_013995921), Trout SelPa2 (MH085053), Salmon SelPa2 (MH085055), Trout SelPb2 (MH085056), Salmon SelPb2 (MH085057), Pike SelPa (XP_012992107), Pike SelPb (XP_010889663), Zebrafish SelPa (AAH63960), Zebrafish SelPb (AAH86844), Stickleback SelPa (ENSGACG00000006838), Stickleback SelPb (DW037066), Tilapia SelPa (XP_013127321), Tilapia SelPb (XP_005475970), Medaka SelPa (XP_004072267), Medaka SelPb (XP_004079176), Fugu SelPa (XP_003974909), Fugu SelPb (XP_011614676), Chicken_SelPa (NP_001026780), Chicken_SelPb (NP_001335698), Crocodile_SelPa (XM_019532012), Crocodile_SelPb (XM_019531330), Clawed frog (Frog) SelPa (NP_001186825), Frog SelPb (NP_001335928) Coelacanth SelPa (XP_006014647), Coelacanth SelPb (XP_006012455), Spotted gar (S-gar) SelPa (XM_015368034), S-gar SelPb XM (006634737), Elephant shark (E-shark) SelPa (XM_007901591), E-shark_SelPa (XM_007899837), Whaleshark_SelPa (XM_020519442), Whaleshark SelPb (XP_020374664), Platypus SelPa (XP (007654576), Platypus SelPb (XP_007664551). The Amphioxus SelPa and SelPb were translated from XM_002598433 and XM_002604219 and reported by Jiang et al. (2012).
