# Supplementary material for: Four selenoprotein P genes exist in salmonids: Analysis of their origin and expression following Se supplementation and bacterial infection
Source: PLoS One. 2018 Dec 20;13(12):e0209381. doi: 10.1371/journal.pone.0209381 (PMC6301783; doi:10.1371/journal.pone.0209381)
Supplement: S1 Table — (DOCX) [file pone.0209381.s008.docx]

**S1 Table: Predicted SECIS sequences and 2D structure for trout and salmon SelP genes as redicted from their mRNA sequences.**

**
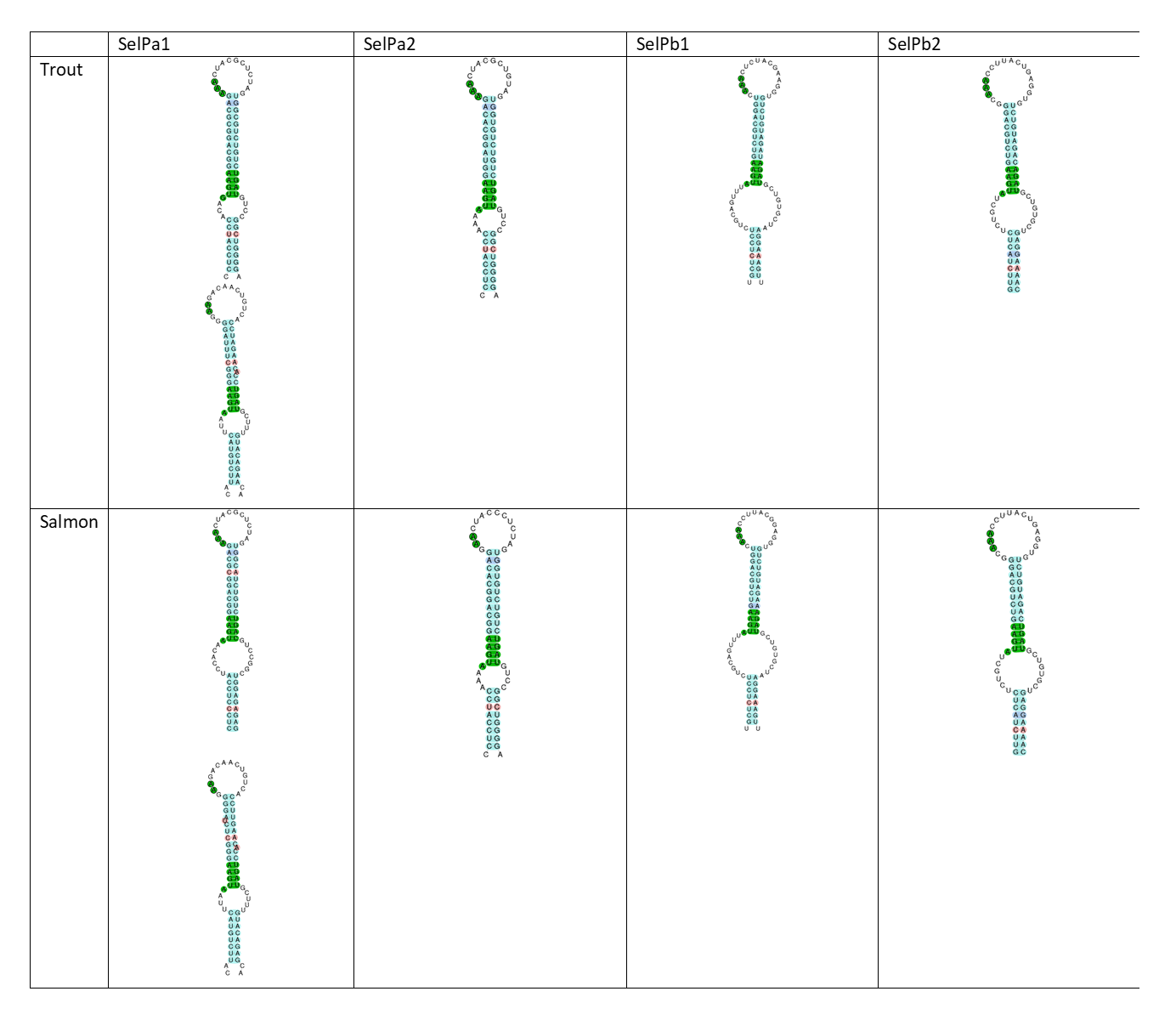
**
