# Supplementary material for: Four selenoprotein P genes exist in salmonids: Analysis of their origin and expression following Se supplementation and bacterial infection
Source: PLoS One. 2018 Dec 20;13(12):e0209381. doi: 10.1371/journal.pone.0209381 (PMC6301783; doi:10.1371/journal.pone.0209381)
Supplement: S2 Table — Amino acid sequence identity (top right) and similarity (bottom left) of vertebrate SelPa and SelPb. The accession numbers are as in S7 Fig. (DOCX) [file pone.0209381.s009.docx]

**S2 Table: Amino acid sequence identity (top right) and similarity (bottom left) of vertebrate SelPa and SelPb. The accession numbers are as in Figure S7.**
